# Prevalence of Undiagnosed Obstructive Sleep Apnea Among Patients Hospitalized for Cardiovascular Disease and Associated In-Hospital Outcomes: A Scoping Review

**DOI:** 10.3390/jcm9040989

**Published:** 2020-04-02

**Authors:** Colin Suen, Jean Wong, Clodagh M. Ryan, Samuel Goh, Tiffany Got, Rabail Chaudhry, Douglas S. Lee, Frances Chung

**Affiliations:** 1 Department of Anesthesiology and Pain Management, University Health Network, Toronto Western Hospital, Toronto, ON M5T 2S8, Canada; colin.suen@mail.utoronto.ca (C.S.); jean.wong@uhn.ca (J.W.); clodagh.ryan@uhn.ca (C.M.R.); samuelgoh@rcai.ie (S.G.); tiffany.got@mail.utoronto.ca (T.G.); rabail.chaudry@mail.utoronto.ca (R.C.); 2 Department of Anesthesia, University of Toronto, Toronto, ON M5G 1E2, Canada; 3 Centre for Sleep Health and Research, Toronto General Hospital, Toronto, ON M5G 2C4, Canada; 4 Division of Cardiology, Peter Munk Cardiac Centre & University Health Network, Toronto, ON M5G 2C4, Canada; douglas.lee@ices.on.ca; 5 Institute for Clinical Evaluative Sciences, Toronto, ON M4N 3M5, Canada

**Keywords:** obstructive sleep apnea, acute coronary syndromes, congestive heart failure, sleep disordered breathing, cardiovascular disease, hospital outcomes

## Abstract

Background: Obstructive sleep apnea (OSA) is associated with long-term cardiovascular morbidity and is highly prevalent in patients with cardiovascular disease (CVD). The objectives of this scoping review were to determine the prevalence of OSA inpatients hospitalized for CVD and to map the range of in-hospital outcomes associated with OSA. Methods: We searched MEDLINE(R), Embase, and Cochrane Databases for articles published from 1946–2018. We included studies involving non-surgical adults with OSA or at high risk of OSA who were hospitalized for CVD. The outcomes were considered as in-hospital if they were collected from admission up to 30 days post-discharge from hospital. Results: After the screening of 4642 articles, 26 studies were included for qualitative synthesis. Eligible studies included patients presenting with acute coronary syndromes (n = 19), congestive heart failure (n = 6), or any cardiovascular disease (n = 1). The pooled prevalence of OSA in cardiac inpatients was 48% (95% CI: 42–53). The in-hospital outcomes reported were mortality (n = 4), length of stay (n = 8), left ventricular ejection fraction (n = 8), peak troponin (n = 7), peak B-type natriuretic peptide (n = 4), and composite cardiovascular complications (n = 2). Conclusions: OSA is highly prevalent in the cardiac inpatient population. The outcomes reported included mortality, cardiac function, cardiac biomarkers, and resource utilization. There are significant knowledge gaps regarding the effect of treatment and OSA severity on these outcomes. The findings from this review serve to inform further areas of research on the management of OSA among patients with CVD.

## 1. Background

Obstructive sleep apnea (OSA) is associated with significant long-term morbidity. OSA is a highly prevalent condition affecting approximately 17–60% of women and 34–84% of men in the general population [1]. Data from observational studies suggest an increased prevalence of OSA in the cardiac patient population [2,3]. Conversely, data from the Sleep Heart Health Study showed that 23.6% of the participants with OSA had coexisting cardiovascular disease, defined as myocardial infarction, angina, coronary revascularization, heart failure, or stroke [4]. Even more concerning is the high prevalence of unrecognized OSA in the cardiac population [5], a subset of the OSA population at higher risk of long-term adverse cardiovascular events compared to diagnosed OSA [6].

Several long-term studies have associated OSA with the incidence of coronary artery disease, left ventricular hypertrophy, hypertension [7], atrial fibrillation [8], pulmonary hypertension [9], cerebrovascular accidents [10], and sudden death [11]. Although the impact of OSA on the development of cardiovascular disease in longitudinal studies is well established, it is uncertain if OSA contributes to acute complications in hospitalized non-surgical patients. Consequently, evidence to guide the management of cardiac inpatients with OSA is limited. Previous studies have highlighted that cardiac inpatients with a high risk of OSA are more likely to experience critical events requiring activation of rapid response systems [12,13]. Treatments such as positive airway pressure (PAP) therapy can potentially reduce critical events, which have several implications for clinical practice [12].

The objective of this scoping review was to identify the prevalence of OSA and to establish outcomes associated with OSA in patients hospitalized for CVD. We have identified the following specific questions:

1. Among adult inpatients (≥18 years old) hospitalized for cardiovascular disease, what is the overall prevalence of OSA?
2. What are the in-hospital outcomes associated with OSA in adult inpatients hospitalized for cardiovascular disease?
3. What is the proportion of patients with OSA who receive treatment while hospitalized for cardiovascular disease?

We used the Arksey and O’Malley methodological framework for the scoping reviews [14]. The goals of this type of review are as defined by Arksey and O’Malley: 1. To examine the extent, range, and nature of research activity. 2. To determine the value for undertaking a full systematic review. 3. To summarize and disseminate research findings. 4. To identify research gaps in the existing literature.

## 2. Methods

### 2.1. Eligibility Criteria

We included studies involving adult (≥18 years of age) inpatients hospitalized for cardiovascular disease with OSA. Examples of cardiovascular disease include but were not limited to acute coronary syndromes (STEMI, NSTEMI, and unstable angina), arrhythmias, and congestive heart failure (CHF). Patients were defined as being diagnosed with OSA or at high risk of OSA based on polysomnography (PSG), screening questionnaires, clinical assessment, chart diagnosis (medical history), or ICD-9 code (administrative/billing records). Patients hospitalized for non-cardiac conditions were excluded. Patients with other sleep disordered breathing syndromes such as central sleep apnea (CSA) were excluded. As well, surgical patients were excluded, since the cardiac surgery population has already been studied in several systematic reviews [15,16].

### 2.2. Study Selection

We included quantitative and qualitative study designs. Studies included, but were not limited to experimental designs (randomized controlled trials, controlled clinical trials, retrospective cohort or case control, and case series). We did not include case reports, editorials, commentaries, or grey literature (i.e. conference abstracts and theses).

### 2.3. Search Strategy

The search strategy was developed in consultation with an information specialist (ME). We searched Ovid MEDLINE(R), Ovid MEDLINE(R) In-Process & Other Non-Indexed Citations, Embase, and Cochrane Databases (1946–2018). The full electronic search strategy is attached (Supplemental File 1). References were limited to English language. In addition to our electronic search, we also performed manual searches of references (hand searching).

### 2.4. Data Extraction

Data were extracted using standardized data collection forms using Distiller SR (Evidence partners, Ottawa, Canada). Screening and data collection were performed by two independent reviewers to minimize reporting bias (TG, SG). Conflicts and disagreements were resolved by a third member of the review team (CS, FC). In-hospital outcomes were collected from admission up to 30 days post-discharge from hospital. Outcomes included: mortality, length of stay, adverse cardiovascular events, cardiac biomarkers, and resource utilization.

### 2.5. Statistical Analysis

Pooled prevalence was determined from studies, which utilized type 1–3 monitoring to establish a diagnosis of OSA, which are acceptable methods as per the American Academy of Sleep Medicine (AASM) consensus guidelines [17]. DerSimonian-Laird random effects models were used to pool estimates of the proportion of OSA. Cochrane Q, visual inspection of forest plots, and the I^2 percentage were used to assess the between-study heterogeneity. The I^2 was extremely high (95.1%), so moderator analyses were conducted to see if this high heterogeneity could be explained by cardiac diagnosis, OSA diagnostic threshold, or OSA diagnostic method. Visualization of the meta-regression by moderators and the omnibus Q test of moderators were used to detect moderating effects. Forest plots were presented by precision (inverse variance) and included Clopper-Pearson confidence intervals, as well as prediction interval for the overall estimate on proportion. This analysis was conducted using meta and metafor packages in R 3.6.0.

## 3. Results

### 3.1. Study Selection

**Figure 1:**
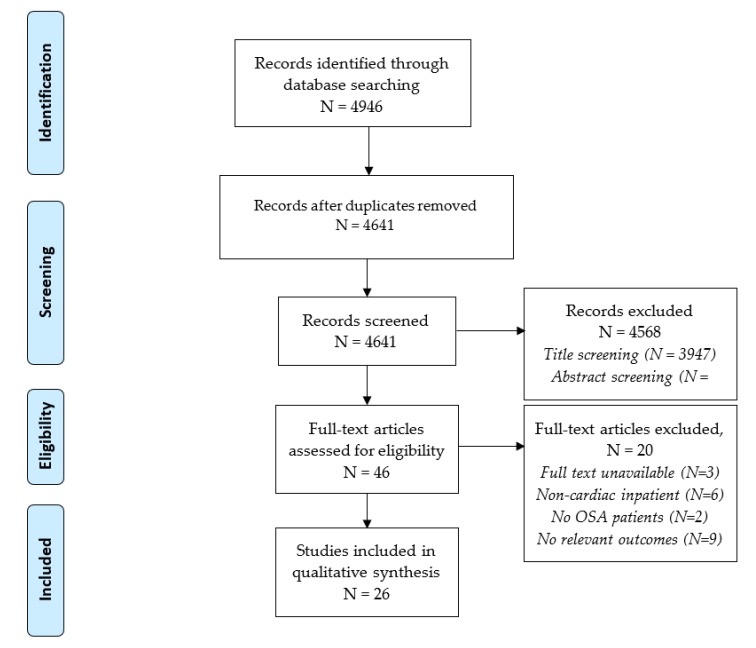
PRISMA study flow diagram.

In total, 4641 studies were identified using our electronic search strategy after eliminating duplicates (Figure 1). After screening, 26 studies were selected for inclusion in the review for qualitative synthesis. The majority of studies involved patients presenting with acute coronary syndromes (ACS; STEMI, NSTEMI, and unstable angina) (n = 19), while 5 studies involved patients presenting with heart failure and 1 study involved patients with any cardiovascular disease such as ACS, CHF, or new arrhythmias. Study and patient characteristics are summarized in Table 1.

### 3.2. Prevalence of OSA Among Hospitalized Cardiac Inpatients

The prevalence of OSA in cardiac inpatients was reported in 24 eligible studies. OSA was diagnosed using different levels of monitoring, as previously described [43]. Type 1 monitoring was defined as in-laboratory, technologist attended PSG (minimum of 7 channels: EEG, EOG, chin EMG, ECG, airflow, respiratory effort, and SpO_2_). Type 2 monitoring is an unattended PSG with a minimum of 7 channel. Type 3 involved portable monitoring (minimum of 4 channels: respiratory movement, airflow, heart rate, SpO2). Type 4 involved portable monitoring with 1 or 2 channels, including pulse oximetry [44].

**Figure 2:**
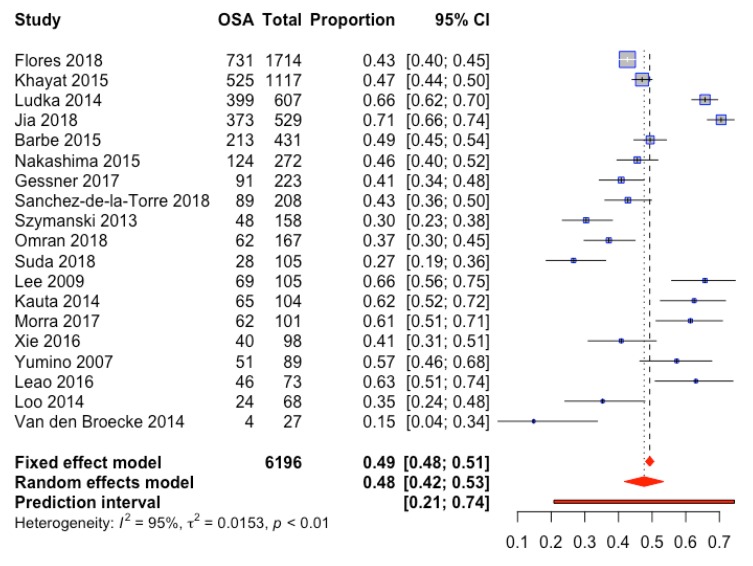
Forrest plot of OSA prevalence among hospitalized cardiac inpatients.

We only estimated the prevalence from studies which utilized type 1–3 sleep studies, with validated scoring criteria and definitions for apnea and hypopnea from AASM [45]. Nineteen studies were included in a meta-analytic random effects model to determine the true prevalence of OSA among cardiac patients (Figure 2). The proportion estimate is 48% (42–53% 95%CI). Due to high heterogeneity (I_2_ 95%), tests for outliers and moderators were conducted but none were found to be significant; no study was found to be outlying and this heterogeneity was not explained by specific cardiac diagnosis (ACS, CHF, or other CVD) (Supplemental Figure S1), diagnostic method, or diagnostic threshold (Supplemental Figure S1). Varying apnea hypopnea index (AHI) thresholds of 5 (n = 9), 10 (n = 1), or 15 (n = 10) events/h for the diagnosis of OSA were reported. The estimated prevalence of OSA for studies using a diagnostic threshold of AHI 15 was 46% (37–54, 95% CI) versus 50% (40–59, 95% CI) in studies using a threshold of AHI 5 or 10. Subgroup analysis revealed an estimated prevalence of 49% (42–56%, 95% CI) for ACS, and 43% (32–55%, 95%CI) for heart failure (HF) and CVD. We consider the prediction interval to be (0.21, 0.74). In other words, if another study were conducted, this is the interval in which the OSA proportion for the next study is expected to lie based on the data available in this study. However, due to the heterogeneity between studies, the interval is wide.

The timing of the sleep studies and AHI are summarized in Table 1. Overall, most studies established a diagnosis of OSA using a sleep study within three days of admission (n = 9). The remainder were either during admission at an unspecified time (n = 6), post discharge (n = 1), greater than 3 days after admission or PCI (n = 2), or not reported (n = 1). The design of these studies involving sleep monitoring was such that the patients included did not have a prior diagnosis of OSA and are therefore newly diagnosed.

Two studies identified OSA patients using nocturnal oximetry (Type 4 monitor), using the oxygen desaturation index (ODI) ≥ 5 as the threshold. ODI is defined as the number of desaturation events with a drop in SpO_2_ of at least 3% or 4% from baseline per hour [45]. The ODI obtained by oximetry has been demonstrated to have good correlation with AHI [46]. Both of these studies consisted of patients with heart failure and reported the proportion of OSA as 41% and 42.9%, respectively. Three other studies reported the prevalence of OSA based on clinical parameters. Mohananey et al. reported a much lower OSA prevalence of 1.3% using administrative data (International Classification of Diseases, Ninth Revision, Clinical Modification ICD-9-CM) [39]. Marin et al. defined OSA as having met 3 criteria: heavy snoring, excessive daytime sleepiness, and having an ODI ≥ 10. Using this definition, the authors reported an OSA prevalence of 21.9% among patients with acute myocardial infarction [41]. Andrechuk et al. used a score of ≥ 2 on the Berlin Questionnaire (BQ) to define a patient at high risk of OSA among patients hospitalized for acute MI and using this score, the prevalence was estimated to be 60% [40].

## 4. In-Hospital Outcomes of Cardiac Patients with OSA

### 4.1. In-Hospital Mortality

The in-hospital outcomes of cardiac inpatients with OSA are reported in Table 1. In-hospital mortality was reported in four studies involving OSA patients presenting with acute coronary syndromes with contrary results. One study reported a statistically significant increase in 30-day mortality in patients with a high suspicion of OSA (7.4 vs. 1%, *p* = 0.03) [25]. While in contrast, OSA was associated with a significant decrease in in-hospital mortality compared to patients without OSA (3.7 vs. 7.4%, respectively, aOR 0.78 *p* < 0.05) [39]. The use of ICD-9-CM coding in the latter study to identify patients with OSA may have led to the inclusion of undiagnosed or untreated OSA in the “non-OSA group”.

### 4.2. Length of Stay

Eight studies reported data on OSA in relation to hospital length of stay (LOS) (Table 2) with six studies reporting trends towards increased LOS in OSA vs. non-OSA patients. Additionally, Barbé et al. reported a significantly increased CCU length of stay in patients with OSA vs. non-OSA (2.6 vs. 2.3 days, respectively, *p* < 0.05), with no difference in overall hospital length of stay.

### 4.3. Composite Cardiac Complications

There were no significant differences in the composite cardiac complications [41,47]. In a study of 251 patients admitted for acute MI, Marin et al. found no significant differences in the proportion of patients who developed major complications following acute MI, defined as shock, congestive heart failure, or cardiac tamponade, between the OSA (38.2%) and non-OSA (34.2%) groups [41]. However, a higher rate of ventricular arrhythmias was reported in the OSA vs. non-OSA group [41]. Similarly, an ancillary study of a randomized trial of continuous positive airway pressure (CPAP) in patients with ACS and OSA (ISAACC) found no significant difference in cardiac complications between patients with OSA (AHI > 15) and no OSA (AHI ≤ 15) [47].

### 4.4. Left Ventricular Ejection Fraction (LVEF)

The left ventricular ejection fraction (LVEF) during hospitalization was reported in eight studies, of which six studies included patients with ACS and one study with HF. Overall, none of these studies reported statistically significant differences in LVEF between OSA patients presenting with ACS. In heart failure patients, OSA was associated with a slight decrease in LVEF [34]. Morra et al reported a statistically significant inverse correlation between the AHI and LVEF [29]. Nakashima et al observed that in ACS patients with equivalent LVEF at baseline, after 21 days, the LVEF was significantly lower in patients with OSA vs. control subjects (52% vs. 59%; *p* < 0.02) [2,21].

### 4.5. Cardiac Biomarkers: Troponin, B-Type Natriuretic Peptide

Cardiac biomarkers during hospitalization such as peak troponin (n = 7) and B-type natriuretic peptide (BNP) (n = 4) levels were also reported. The majority of these studies showed no difference between cardiac biomarker levels in OSA and non-OSA patients (Table 2). One study reported increased peak troponin with OSA with ACS, which was correlated with increasing severity of OSA through regression modeling, while another reported lower peak troponin levels in OSA patients with ACS [28,42]. The peak BNP was significantly higher in one study among patients with high (153.2 ± 153.2 pg/mL) vs. low suspicion (22.2 ± 22.2 pg/mL) of OSA based on the Berlin Questionnaire [25].

### 4.6. Resource Utilization

One retrospective cohort study reported on resource utilization [39]. Greater hospital charges were incurred in OSA vs. non-OSA patients presenting with ST-elevation myocardial infarction ($79,460.12 ± 70,621.91 vs. $62,889.91± 69,124.15, respectively, *p* < 0.001) (Table 2) [39].

### 4.7. CPAP Usage

Data on treatment with CPAP was reported in five studies (Table 3). The included studies defined compliance as the usage of treatment for at least four hours per night on at least 70% of nights. Leao et al. reported a compliance rate of 41% at a median 75-month follow-up period. Another study examined the short-term outcomes of hospitalized HF patients with OSA, who were subsequently offered CPAP in hospital, of which 62% continued upon discharge [33]. Of these patients prescribed CPAP, the compliance at 30-day follow-up was 45%. Compliance was associated with a reduced 30-day hospital readmission for cardiac issues compared to those who were non-compliant with CPAP (partial and no usage) [29].

## 5. Discussion

### 5.1. Prevalence of OSA Among Hospitalized CVD Patients

The present scoping review identifies that OSA is a common comorbid condition with an estimated pooled prevalence of 48% among patients hospitalized with cardiovascular disease. Our data suggest that OSA is highly prevalent in hospitalized patients with cardiovascular disease. Furthermore, since all studies included in the meta-analysis established the diagnosis after hospital admission, this proportion may estimate the true prevalence of *undiagnosed* OSA. As a comparison, a recent systematic review and meta-analysis of 32 studies of ACS patients reported a pooled prevalence of sleep-disordered breathing (SDB) at 69%, 43%, and 25% using diagnostic thresholds of AHI > 5, 15, 30 events per hour, respectively [49]. In contrast to our analysis, these studies did not distinguish between OSA and CSA, and therefore contribute to a higher estimate of SDB.

Several clinical questionnaires have been developed to screen for OSA, and in this review, we included a small proportion of studies which established a diagnosis based on clinical information. One study used the Berlin Questionnaire, which has a sensitivity and specificity of approximately 0.76 and 0.59 for mild and 0.77 and 0.44 for moderate OSA, respectively [50]. We observed a large discrepancy between the OSA prevalence reported using ICD-9-CM administrative coding (1.3%) compared to PSG [39]. The accuracy of ICD coding to identify patients with OSA has been criticized as suboptimal as it demonstrated poor sensitivity (58%) and specificity (38%) [51]. The ICD coding system is a means for processing physician billing claims which relies on the physician to recognize and document the diagnosis, which is biased towards underreporting. When compared to other reports using the gold standard method, PSG, which uses objective criteria (AHI > 5) to diagnose OSA, it is very likely that those identified as non-OSA by ICD coding include a significant proportion of patients with undiagnosed OSA, a subgroup which may be at the highest risk of developing in-hospital complications [12]. The overall impact is unclear, but it has been suggested that false negatives would bias the data towards the null, leading to an underestimate of the effect of OSA [52]. For these reasons, we did not include studies using only clinical information in our analysis of pooled prevalence.

### 5.2. Reported In-Hospital Outcomes of OSA Patients Hospitalized for Cardiovascular Disease

In summary, we identified several hospital outcomes associated with OSA: mortality, length of stay, composite cardiovascular complications, left ventricular ejection fraction, peak troponin levels, and peak BNP levels. The current study shows conflicting data regarding mortality and cardiovascular morbidity among cardiovascular inpatients with OSA. The association between the incidence of cardiovascular risk factors such as hypertension, diabetes, and dyslipidemia, and diseases such as coronary artery disease, heart failure, and atrial fibrillation and OSA are well established [53]. In fact, a recent systematic review and meta-analysis found that OSA was associated with an increased risk of major adverse cardiovascular events (MACE) after PCI (RR 1.96) [54]. Furthermore among patients with ACS, OSA was associated with a higher rate of restenosis after PCI at six months repeat revascularization in patients undergoing PCI [31,54].

In the general population, several studies have demonstrated a survival benefit in hospitalized patients with OSA in regular wards and critical care [13,55]. In recent years, OSA-induced chronic intermittent hypoxia has been hypothesized to induce ischemic preconditioning [56,57,58]. Large databases have demonstrated decreased mortality following MI among patients with OSA vs. non-OSA [39], and inverse correlations between AHI and peak troponin levels [59]. Additionally, coronary angiography of patients with OSA has demonstrated increased collateralization around areas of total coronary occlusion [60]. Another explanation is the “obesity paradox”, which is derived from observations of improved cardiovascular prognosis in obese patients compared to their lean counterparts [61]. Several mechanisms, including higher levels of atrial natriuretic peptides, attenuated sympathetic nervous system and renin-angiotensin responses, and higher circulating levels of lipoproteins which may be cardioprotective have been suggested [58]. Epidemiological data have shown that although the overall incidence of MI is higher in obese individuals, the prognosis following MI is improved [62].

### 5.3. OSA Treatment During Hospitalization

Only 4.8–5.8% of hospitalized patients with diagnosed sleep apnea are provided CPAP therapy in hospital [63,64]. CPAP usage was reported in five studies; however, it would not be accurate to estimate usage patterns using these data due to the lack of reporting on CPAP prescribing parameters and reasons for non-compliance. In-hospital compliance, as defined by usage >4 h/night for >70% of nights, is also poorly estimated for several reasons. With the exception of two studies, CPAP was prescribed several days or weeks after hospital admission to allow for an outpatient CPAP titration study. The two studies reported compliance at 41% and 45%, respectively [19,33]. These compliance data were obtained after at least 30 days from admission, well beyond the hospitalization period and therefore, do not represent true in-hospital compliance.

In this review, there was no available data associating *in-hospital* outcomes with CPAP use, representing a significant knowledge gap that needs to be addressed. The RICCADSA randomized controlled trial of CPAP treatment for revascularized coronary artery disease patients showed no difference in the primary endpoint of repeat revascularization, myocardial infarction, stroke, or cardiovascular mortality, but a significant decrease in patients who were CPAP adherent vs. non-adherent [65]. In patients with heart failure, there is evidence to suggest that positive airway pressure (PAP) improves the left ventricular ejection fraction and exercise capacity [66]. Randomized controlled trials testing the effect of CPAP therapy in heart failure with a reduced ejection fraction (EF) show an improved EF with treatment [67,68,69]. Kauta et al performed a study in which heart failure patients underwent PSG and CPAP/BPAP treatment during hospitalization and after discharge for OSA, and found that 5 of 17 (29%) patients who did not use CPAP or were poorly compliant (30%) versus none of the compliant CPAP users were readmitted to the hospital or visited the emergency department for a cardiac issue within 30 days from discharge (*p* = 0.025) [33].

### 5.4. Future Directions

This study illustrates the limitations of current studies and knowledge gaps concerning the outcomes of hospitalized inpatients. Emerging technologies such as portable sleep monitoring and oximetry are improving the accessibility of OSA diagnosis, which should spur new research on hospitalized patients. Sharma et al. proposed a clinical pathway for the rapid screening of OSA using overnight pulse oximetry during hospital admission for heart failure [70]. Follow-up PSG was performed at four weeks post discharge, which showed strong agreement between the oxygen desaturation index (ODI) obtained by oximetry and PSG-derived AHI [70]. As overnight oximetry continues to be validated as a diagnostic tool for OSA, future studies could capitalize on rapid screening to fast track the initiation of treatment such as positive airway pressure therapy while in hospital. Rapid diagnosis and treatment have been demonstrated to be feasible and effective in reducing AHI and hypoxemia using auto-titrating CPAP introduced on the first postoperative night in surgical patients [71]. The disadvantage to early diagnosis is the possibility of false positives, as SDB may be transiently worsened during the acute phase following ACS, followed by normalization two weeks after the event [72]. The reasons for this are unclear, but could be due to rostral fluid shifts in patients with predisposing factors such as CHF leading to pharyngeal collapsibility [73]. Therefore, the ideal timing for OSA diagnosis is an area which may require further investigation.

Future prospective studies specifically designed to detect the differences between in-hospital outcomes in OSA vs. non-OSA patients are needed. Study designs should also incorporate sleep apnea staging or severity as a factor for risk stratification. Patients with untreated OSA may potentially benefit from increased monitoring by the means of continuous pulse oximetry and/or capnography. A recent systematic review and meta-analysis of adult surgical patients who were prescribed opioids showed that receiving continuous pulse oximetry improved the detection of oxyhemoglobin desaturation and there was a trend towards less ICU transfers when compared to intermittent nursing spot checks [74]. Unfortunately, there is limited literature on the efficacy of in-hospital treatment of OSA using positive airway pressure therapy. These data are urgently needed in order to inform the management of hospitalized OSA patients.

### 5.5. Limitations

Our scoping review has several limitations. There was considerable heterogeneity between the studies such as type of monitoring equipment, definitions for hypopnea, AHI cut-offs, and patient characteristics. Ideally, the prevalence should be compared to a control population within the same study, however, none of the included studies provided a reference control population without cardiovascular disease. Our primary focus was to chart the outcomes related to the hospitalization of patients with cardiovascular disease, and therefore, the study outcomes were primarily cardiac in nature. Other potential outcomes of interest related to OSA such as respiratory complications and neurological complications were not reported but may have clinical relevance. This scoping review does not evaluate the quality of evidence. This review is primarily qualitative, and therefore, does not make any definitive conclusions on the directionality of the data. 

## 6. Summary and Conclusions

OSA is highly prevalent in the cardiac inpatient population. The outcomes reported included mortality, cardiac function, cardiac biomarkers, and resource utilization. There are significant knowledge gaps regarding the effect of treatment and OSA severity on these outcomes. The findings from this review serve to inform further areas of research on the management of OSA among patients with CVD.

## Figures and Tables

**Table 1:** Study and patient characteristics.

Reference/Country	Study Design/CVD Dx	OSA Dx Method/ Threshold	Timing of OSA Testing	Subgroup, n (%)	AHI
Gessner 2017 [18]	Retrospective Cohort	Type 2	≤3 d post admission	OSA, 91 (41)	23
Germany	ACS	AHI ≥ 5		non-OSA, 132 (59)	NR
Leao 2016 [19]	Prospective Cohort	Type 1	55 (31–77) d post admission	OSA, 46 (63)	30.6 ± 23.0
Portugal	ACS	AHI ≥ 5		non-OSA, 27 (37)	2.3 ± 3.2
Barbé 2015 [20]	RCT (ancillary)	Type 3	≤48 h post admission	OSA, 213 (49)	30.6 ± 14.4
Spain	ACS	AHI > 15		non-OSA, 218 (51)	6.4 ± 4.2
Nakashima 2015 [21]	Prospective cohort	Type 1	prior to discharge	OSA, 124 (46)	NR
Japan	ACS	AHI ≥ 15		non-OSA, 148 (54)	NR
Nakashima 2006	Prospective cohort	Type 1	14–21 d post admission	OSA, 37 (43)	31.7 ± 13.6
Japan [22]	ACS	AHI ≥ 15		non-OSA, 49 (57)	5.8 ± 4.2
Van den Broecke 2014 [23]	Prospective cohort	Type 2	≤48 h post admission	OSA, 4 (15)	24.7 ± 19.5
France	ACS	AHI ≥ 15		non-OSA, 23 (85)	2.5 ± 1.9
Loo 2014 [24]	Prospective cohort	Type 3	30 d post discharge	OSA, 24 (35)	24.0 (16.9–52.0)
France	ACS	AHI ≥ 15		non-OSA, 44 (65)	4.9 (0.3–14.9)
Szymanski 2013 [25]	Prospective cohort	Type 3	during hospitalization	OSA, 48 (30)	NR
Poland	ACS	AHI ≥ 5		non-OSA, 109 (70)	NR
Lee 2009 [3]	Prospective cohort	Type 2	between day 2–5 post PCI	OSA, 69 (66)	38.1
Singapore	ACS	AHI ≥ 15		non-OSA, 36 (34)	8.5
Jia 2018 [26]	Prospective Cohort	Type 2	48–72 h post admission	OSA, 373 (70)	37.2 ± 16.9
China	ACS	AHI > 15		non-OSA, 159 (30)	9.4 ± 3.4
Flores 2018 [27]	RCT (ancillary)	Type 3	24–72 h post admission	OSA, 731 (43)	34 (4.4–51.3)
Spain	ACS	AHI ≥ 5		non-OSA, 983 (57)	NR
Sanchez-de-la-Torre 2018 [28]	Prospective cohort (ancillary)	Type 3	24–72 h post admission	OSA, 89 (43)	32.0 (20.8–46.6)
Spain	ACS	AHI ≥ 15		non-OSA, 119 (57)	4.8 (1.6–9.6)
Morra 2017 [29]	Prospective cohort study	Type 3	24–72 h post admission	OSA, 62 (61)	NR
France	ACS	AHI ≥ 5		non-OSA, 39 (39)	NR
Xie 2016 [30]	Prospective cohort	Type 2	median 7 day post MI	OSA, 40 (41)	42.5 (33.1–52.6)
USA	ACS	AHI ≥ 15		non-OSA, 58 (59)	30.0 (20.5–41.6)
Yumino 2007 [31]	Prospective cohort	Type 3	7–14 d post PCI	OSA, 51 (57)	20.2 ± 10.9
Japan	ACS	AHI ≥ 10		non-OSA, 38 (43)	5.0 ± 3.2
Ludka 2014 [32]	Prospective cohort	Type 3	≥48 h post admission	OSA, 399 (66)	NR
Czech Republic	ACS	AHI≥5		non-OSA, 208 (34)	NR
Kauta 2014 [33]	Prospective cohort	Type 3	during hospitalization	OSA, 65 (63)	24.7 ± 19.5
USA	CVD	AHI ≥ 5		non-OSA, 39 (37)	2.5 ± 1.9
Khayat 2015 [34]	Prospective cohort	Type 2	during hospitalization	OSA, 525 (47)	36 ± 16
USA	HF	AHI ≥ 15		non-OSA, 592 (53)	9.5 ± 4.3
Omran 2018 [35]	Retrospective cohort	Type 3	during hospitalization	OSA, 62 (37)	31.6 ± 15.9
Germany	HF	AHI ≥ 15		non-OSA, 105 (63)	8.8 ± 3.4
Suda 2018 [36]	Prospective cohort	Type 3	3 d (median) after initial clinical improvement	OSA, 28 (27)	NR
Japan	HF	AHI ≥ 5		non-OSA, 77 (73)	NR
Arikawa 2009 [37]	Prospective cohort	Type 4	during hospitalization	OSA, 21 (43)	NA
Japan	HF	ODI ≥ 5		non-OSA, 28 (57)	NA
Ohmura 2014 [38]	Prospective cohort	Type 4	after clinical improvement	OSA, 41 (41)	NA
Japan	HF	ODI ≥ 5		non-OSA, 59 (59)	NA
Mohananey 2017 [39]	Retrospective cohort	Chart review	NA	OSA, 24623 (1.3)	NA
United States	ACS	ICD-9CM 327.23		non-OSA, 1826002 (98.7)	NA
Andrechuk 2016 [40]	Prospective cohort	BQ	≤72 h post admission	OSA, 68 (60)	NA
Brazil	ACS	BQ+ ≥ 2 categories		non-OSA, 45 (40)	NA
Marin 1998 [41]	Prospective cohort	Clinical+oximetry	within 24 h post admission	OSA, 55 (22)	NA
Spain	ACS	heavy snorers, reported EDS, ODI > 10		non-OSA, 196 (78)	NA
Sommerfeld 2017 [42]	Prospective cohort	Chart review	NA	OSA, 99 (29)	NA
USA	HF			non-OSA, 245 (61)	NA

Abbreviations: AHI = apnea hypopnea index, ACS = acute coronary syndromes, BQ = Berlin Questionnaire, CVD = cardiovascular disease, HF = heart failure, Dx = diagnosis, ESS = Epworth Sleepiness Scale, EDS = excessive daytime sleepiness, ICD-9CM = International Classification of Diseases, ninth revision, Clinical Modification, NA = not applicable NR = not reported, ODI = oxygen desaturation index, RCT = randomized controlled trial. Data expressed as mean ± SD unless otherwise stated or median (interquartile range) unless otherwise stated.

**Table 2:** In-hospital outcomes of patients with OSA hospitalized for CVD.

Reference	Cardiac Dx	Outcome OSA	Outcome Non-OSA/Control	Comments
**In-Hospital Mortality**				
Mohananey 2017 [39]	ACS	3.7%	7.4%	aOR, 0.83 (95% CI, 0.81–0.84); *p* < 0.001
Barbe 2015 [20]	ACS	0.70%	0%	*p* = 0.29, ns
Szymanski 2013 [25]	ACS	7.40%	1%	*p* = 0.03. High vs. low suspicion of OSA
Marin 1998 [41]	ACS	12.7%	10.2%	ns
**Length of Stay (days**)				
Mohananey 2017 [39]	ACS	5 ± 4.68	4.85 ± 5.96	*p* < 0.001
Leao 2016 [19]	ACS	5.5 (IQR 5–9)	7 (IQR 3.8–7.5)	*p* = 0.292, ns
Khayat 2015 [34]	HF	9 ± 11.4	7.2 ± 8	*p* < 0.05
Barbe 2015 [20]	ACS	6.8 ± 3.8 CCU: 2.6 ± 1.3	6.5 ± 3.7 CCU: 2.3 ± 1.0	ns *p* < 0.05
Szymanski 2013 [25]	ACS	10.4 ± 5.2	8.7 ± 4	*p* = 0.016. High vs. low suspicion of OSA
Jia 2018 [26]	ACS	8 ± 5.6	6.7 ± 4.2	*p* = 0.007
Sommerfeld 2017 [42]	HF	6.2 ± 5.9	5.5 ± 4.8	*p* = 0.235
Ohmura 2014 [38]	HF	15 ± 10	16 ± 10	*p* = 0.804
**Left Ventricular Ejection Fraction (LVEF)**				
Gessner 2017 [18]	ACS	50 ± 12%	57 ± 7%	*p* = n/a
Khayat 2015 [34]	HF	26.3 ± 10.5%	29.5 ± 10.4%	*p* < 0.05
Barbe 2015 [47]	ACS	54.8 ± 11.6%	57 ± 9.5%	ns. OSA associated with LVEF < 51.5% (OR 2.05, *p* = 0.04)
Leao 2016 [19]	ACS	49.4 ± 9.2%	51.2 ± 8.7	*p* = 0.462, ns
Loo 2014 [24]	ACS	52 ± 13.9	52 ± 11.4	*p* = 0.989
Morra 2017 [29]	ACS	51.5 ± 6.2	52.7 ± 6.4	ns
Nakashima 2006 [22]	ACS	Baseline: 54 ± 12 Day 21: 52 ± 12	53 ± 12 59 ± 13	ns *p* = 0.022
Ohmura 2014 [38]	HF	28±10	30 ± 10	*p* = 0.444
**Cardiovascular Complications**				
Barbe 2015 [47]	ACS	8.1%	9.8%	Ns
Marin 1998 [41]	ACS	38.2%	34.2%	Ns. Rate of ventricular arrhythmias higher in OSA vs. non-OSA
**Peak Troponin**				
Gessner 2017 [18]	ACS	37791 ± 52652 ng/L	5368 ± 4357 ng/L	*p* = n/a
Leao 2016 [19]	ACS	27.7 ± 36.3 ng/mL	28 ± 34.8 ng/mL	*p* = 0.974, ns
Loo 2014 [24]	ACS:	54 (IQR 7.8–80.0) ug/L	80.0 (IQR 0.3–80.0) ug/L	0.345, ns
Jia 2018 [26]	ACS	9.7 ± 9.7 ng/mL	8.3 ± 8.3 ng/mL	*p* = 0.534
Sanchez-de-la-Torre 2018 [28]	ACS	3.79 (IQR 0.37–243) ng/mL	10.70 (1.78–40.1) ng/mL	*p* = 0.04. Higher # stents placed in OSA vs. non-OSA
Morra 2017 [29]	ACS	3685 ± 3576 ng/L	2830 ± 3333 ng/L	*p* = 0.08
Barbe 2015 [47]	ACS	987.2 ± 884.9 ng/L	831.7 ± 908. 4 ng/L	*p* = 0.002, Regression modelling shows increased peak Troponin with increasing AHI
**Peak BNP**				
Gessner 2017 [18]	ACS	241 ± 308 pg/ml	177 ± 261 pg/ml	*p* = n/a
Szymanski 2013 [25]	ACS	153.2 ± 153.2 pg/mL	22.2 ± 22.2 pg/mL	*p* = 0.0001. High vs. low suspicion of OSA
Jia 2018 [26]	ACS	90.8 ± 240.1 pg/mL	60.3 ± 139 pg/mL	*p* = 0.068
Ohmura 2014 [38]	HF	206 ± 206	147 ± 138	*p* = 0.101
**Resource utilization**				
Mohananey 2017 [39]	ACS	$79 460.12 ± 70 621.91	$62 889.91 ± 69 124.15	*p* < 0.001

Abbreviations: OSA—obstructive sleep apnea, ACS—acute coronary syndromes, HF—heart failure, aOR—adjusted odds ratio, ns—non-significant, ICD-9CM—International Classification of Diseases, Ninth Edition, Clinical Modification, BQ = Berlin Questionnaire, ESS = Epworth Sleepiness scale.

**Table 3:** Proportion of patients receiving positive airway pressure therapy for OSA.

Reference	% CPAP Use	Compliance (%) *	Findings
**Leao 2016 [19]**	100 (study design)	41	PSG and CPAP prescribed after clinical stabilization. CPAP compliant group associated with fewer CV events and less severe ones at 75 months follow-up (RR 0.87, 95% CI 0.31 to 2.46, *p* = 0.798).
**Nakashima 2015 [21]**	59	NR	After admission – PSG and CPAP prescribed. Mean follow-up duration 4.4 yrs. Although CPAP treatment decreased the incidence of ACS recurrence and MACEs, these differences were not significant (9% vs. 23%, *p* = 0.056; 14% vs. 31%, *p* = 0.053, respectively). Similarly, ACS- and MACE-free survival estimates were not different between patients with and without CPAP treatment (log rank *p* = 0.129; *p* = 0.129, respectively).
**Nishihata 2015 [48]**	49	NR	Retrospective study of patients with PSG after hospitalization for CVD. CPAP vs. no-CPAP compared. Nightly CPAP use 5.0 ± 1.7 h
**Kauta 2014 [33]**	62	45	In-hospital PSG, followed by 50 patients prescribed CPAP after admission. Decreased proportion of 30-day hospital readmission for HF in compliant CPAP users vs. non-compliant.
**Xie 2016 [30]**	8.2	NR	CPAP users excluded from analysis

* Compliance defined as >4 h of nightly use, >70% of nights.

## Supplementary Materials

The following are available online at https://www.mdpi.com/2077-0383/9/4/989/s1. Supplementary File S1: Electronic search strategy. Supplementary Figure S1. Forrest plots of OSA prevalence among cardiac inpatients by subgroups according (A) Cardiac diagnosis and (B) AHI thresholds for OSA diagnosis.

## Abbreviations

ACS	acute coronary syndromes
AHI	apnea hypopnea index
BNP	b-type natriuretic peptide
CHF	congestive heart failure
CPAP	continuous positive airway pressure
CSA	central sleep apnea
CVD	cardiovascular disease
HF	heart failure
ICD-CM-9	international classification of diseases, clinical modification 9th edition
LOS	length of stay;
LVEF	left ventricular ejection fraction
MACE	major adverse cardiovascular events
NSTEMI	non-ST elevation myocardial infarction
OSA	obstructive sleep apnea
PAP	positive airway pressure
SDB	sleep-disordered breathing
STEMI	ST-elevation myocardial infarction
