# Supplementary material for: Prevalence of Undiagnosed Obstructive Sleep Apnea Among Patients Hospitalized for Cardiovascular Disease and Associated In-Hospital Outcomes: A Scoping Review
Source: J Clin Med. 2020 Apr 2;9(4):989. doi: 10.3390/jcm9040989 (PMC7230766; doi:10.3390/jcm9040989)
Supplement: Supplementary file 1 [file jcm-09-00989-s001.pdf]

Data Supplement

Supplementary File 1: Electronic search strategy

Obstructive Sleep Apnea + Cardiac or Cardiovascular Disease/PCI + Inpatients; limited to human, adults, English language where possible

The databases searched were:

1. Medline; 2. Medline In-Process/ePubs; 3. Embase; 4. CCTR; 5. CDSR; 6. PubMed-NOT-Medline;

Medlinem

Ovid MEDLINE(R) <1946 to May Week 3 2018>

#	Searches	Results
1	exp Sleep Apnea Syndromes/ [MeSH heading changed from exp sleep disordered breathing]	31038
2	exp obesity hypoventilation syndrome/	750
3	apnea/ and sleep/ [historical]	263
4	apnea/ and (hypoventilation/ or sleep disorders/) [historical]	213
5	(apn?ea adj1 attack*).mp.	35
6	(apn?ea adj1 index*).mp.	921
7	(apn?ea adj1 indices*).mp.	63
8	(apn?ea adj1 monitor*).mp.	272
9	(haddad* adj1 syndrom*).mp.	25
10	(high* altitud* adj2 (period* adj2 breath*)).mp.	10
11	(hypersomni* adj2 periodic breath*).mp.	11
12	(hypersomni* adj2 periodic respirat*).mp.	8
13	(nocturnal adj2 hypoventilation).mp.	195
14	(nocturnal adj2 hypoxemia).mp.	375
15	(obes* hypoventil* adj2 apne*?).mp.	30
16	(obes* hypoventil* adj2 apnoe*?).mp.	10
17	(obstruct* adj2 hypoapnea*).mp.	0
18	(obstruct* adj2 hypo-apnea*).mp.	0
19	(obstruct* adj2 hypoapnoea*).mp.	0
20	(obstruct* adj2 hypo-apnoea*).mp.	0
21	(obstruct* adj2 hypopnea*).mp.	330
22	(obstruct* adj2 hypopnoea*).mp.	60
23	(ondine* adj2 curse*).mp.	214
24	(ondine* adj2 syndrome*).mp.	80
25	(pickwick* adj1 syndrom*).mp.	333
26	(sleep adj2 respirat* adj1 disorder*).mp.	253
27	(sleep disorder* adj1 breathing).mp.	5052
28	(sleep disorder* adj1 respirat*).mp.	64
29	(sleep* adj2 hypopn?ea*).mp.	1999
30	(upper airway resistan* adj2 syndrom*).mp.	224

31	apnea-hypopnea*.mp.	6536
32	apneic*.mp.	2352
33	apnoea-hypopnoea*.mp.	1180
34	apnoeic*.mp.	703
35	hypopneic*.mp.	71
36	hypopnoeic*.mp.	13
37	obes* hypoventil* syndrome?.mp.	934
38	osa.tw,kw.	9101
39	osahs.tw,kw.	1108
40	osas.tw,kw.	3384
41	sleep apne*.mp.	34341
42	sleep apnoe*.mp.	4806
43	upper airway* resistan* syndrom*.mp.	232
44	or/1-43 [~~ Obstructive Sleep Apnea & Related Terms ~~]	40189
45	exp Cardiovascular Diseases/	2190328
46	"high blood pressur*".mp.	12001
47	(atrial adj1 fibrillat*).mp.	59964
48	(atrial adj1 flutter*).mp.	7268
49	(cardiac* adj3 disease*).mp.	24001
50	(cardiac* adj3 fail*).mp.	16045
51	(cardiac* adj3 infarc*).mp.	5630
52	(cardiac* adj3 illness*).mp.	461
53	(cardio* adj3 disease*).mp.	216725
54	(cardio* adj3 fail*).mp.	6993
55	(cardio* adj3 illness*).mp.	685
56	(coronar* adj3 disease*).mp.	229292
57	(coronar* adj3 fail*).mp.	2662
58	(coronar* adj3 illness*).mp.	110
59	(coronar* adj3 syndrom*).mp.	27297
60	(heart? adj1 attack*).mp.	4503
61	(heart? adj1 failure*).mp.	162661
62	(heart? adj3 aneurysm*).mp.	7217
63	(heart? adj3 arrest???).mp.	34785
64	(heart? adj3 defect*).mp.	74696
65	(heart? adj3 disease?).mp.	229309
66	(heart? adj3 failure?).mp.	163747
67	(heart? adj3 infarc*).mp.	9547
68	(heart? adj3 isch?em*).mp.	40705
69	(heart? adj3 neoplas*).mp.	15515
70	(heart? adj3 ruptur*).mp.	3330
71	(myocard* adj3 disease*).mp.	8511
72	(myocard* adj3 isch?emi*).mp.	66538
73	(myocard* adj3 infarc*).mp.	213949

74	(myocard* adj3 stunning?).mp.	2789
75	(pericard* adj3 effusion*).mp.	11828
76	(peripheral* adj1 arter* adj2 disease*).mp.	14060
77	(pulmonary adj2 emboli*).mp.	46263
78	(ventricular adj3 outflow* adj3 obstruct*).mp.	4930
79	(ventricular adj2 dysfunct*).mp.	41263
80	angina??.mp.	64661
81	arrhythmi*.mp.	122722
82	arteriosclero*.mp.	71521
83	artherosclero*.mp.	197
84	bradyarrhythmi*.mp.	1549
85	brady-arrhythmi*.mp.	61
86	cardiomegal*.mp.	24705
87	cardio-megal*.mp.	1
88	cardiomyopath*.mp.	79399
89	cardio-myopath*.mp.	55
90	cardiovascular.mp.	453852
91	coronary.mp.	433199
92	CVD.tw,kw.	22335
93	endo-cardi*.mp.	27
94	endocardi*.mp.	50776
95	hyperlipidaemi*5.mp.	4106
96	hyperlipidemi*5.mp.	38736
97	hypertension.mp.	411191
98	hypertensive?.mp.	100158
99	myocardial*.mp.	386434
100	myocardiac*.mp.	316
101	NSTEMI.mp.	1493
102	sick sinus*.mp.	3332
103	STEMI.mp.	7137
104	tachycardi*.mp.	67387
105	(unstab* adj2 angina?).mp.	15119
106	(valvular adj3 heart adj3 disease?).mp.	4953
107	vasculopath*.mp.	8324
108	(left adj1 ventric* adj1 eject* adj1 fraction*).mp.	20779
109	lvef.mp.	9623
110	exp percutaneous coronary intervention/	46591
111	angioplasty, balloon, coronary/	34529
112	atherectomy, coronary/	1604
113	(percutaneous* adj1 coronary adj1 intervention?).mp.	27560
114	(percutaneous* adj1 coronary adj1 revasculari*).mp.	539
115	(coronary adj1 balloon? adj1 angioplast*).mp.	34645
116	(percutaneous* adj1 transluminal* adj1 coronary adj1 angioplast*).mp.	6479

117	(transluminal* adj1 coronary adj1 balloon dilat*).mp.	3
118	(percutaneous* adj1 balloon? adj1 valvuloplast*).mp.	250
119	(coronary adj1 atherectom*).mp.	1833
120	(rotational* adj1 atherectom*).mp.	641
121	or/45-120 [~~ Cardiac or Cardiovascular Diseases or PCI ~~]	2667905
122	44 and 121 [OSA + (Cardiac or CV Diseases or PCI)]	11412
123	Inpatients/	18089
124	exp Hospitalization/ or "length of stay"/	207378
125	Patient admission/ or patient discharge/ or patient handoff/ or patient readmission/ or patient transfer/	62886
126	intensive care units/ or coronary care units/	50443
127	Critical Care/	46895
128	Critical Illness/	23729
129	exp Life Support Care/	8464
130	inpatient?.mp.	87622
131	hospitalise?.mp.	9030
132	hospitalising.mp.	15
133	hospitalisation?.mp.	13100
134	hospitalize?.mp.	81163
135	hospitalizing.mp.	131
136	hospitalization?.mp.	169127
137	inhospital.mp.	1392
138	"in hospital".ab. /freq=2	15407
139	(hospital? adj6 (stay or stays or stayed or staying)).mp.	74850
140	(length* adj6 (stay or stays or stayed or staying)).mp.	103639
141	(patient? adj6 (stay or stays or stayed or staying)).mp.	25713
142	exp Hospital Departments/	168424
143	Patient Care/	9036
144	Continuity of Patient Care/	17381
145	Subacute Care/	853
146	(acute adj1 care adj3 unit?).mp.	751
147	(acute?? adj2 ill*).mp.	11067
148	(coronary care adj3 unit?).mp.	6670
149	(critical care adj3 unit?).mp.	2627
150	(intensive care adj3 unit?).mp.	110790
151	(subacute?? adj2 ill*).mp.	70
152	(ward or wards).mp.	45445
153	(cardiac care adj3 unit?).mp.	452
154	CICU.mp.	143
155	CVICU.mp.	42
156	ICU.mp.	38180
157	MSICU.mp.	7
158	TNICU.mp.	1
159	(admit or admits or admitted or admitting).mp.	159732

160	(admission or admissions).mp.	176211
161	readmit*.mp.	5001
162	readmis*.mp.	21747
163	(critical?? adj2 ill*).mp.	44929
164	(acute adj2 care).mp.	20102
165	exp Hospitals/	251894
166	exp Hospital Units/	97387
167	patient.ab. /freq=2 or patients.ab. /freq=2 or (patient or patients).ti.	3724636
168	(hospital* or department? or ward or wards or room or rooms or care unit or care units or undergo*).mp.	1992266
169	167 and 168	862404
170	or/123-166,169	1571285
171	122 and 170	1563
172	limit 171 to english language	1392
173	limit 172 to humans	1388
174	limit 173 to ("all infant (birth to 23 months)" or "all child (0 to 18 years)" or "newborn infant (birth to 1 month)" or "infant (1 to 23 months)" or "preschool child (2 to 5 years)" or "child (6 to 12 years)" or "adolescent (13 to 18 years)")	228
175	173 not 174	1160
176	limit 173 to ("all adult (19 plus years)" or "young adult (19 to 24 years)" or "adult (19 to 44 years)" or "young adult and adult (19-24 and 19-44)" or "middle age (45 to 64 years)" or "middle aged (45 plus years)" or "all aged (65 and over)" or "aged (80 and over)")	1115
177	175 or 176	1282
178	remove duplicates from 177	1268

Medline In-Process

Ovid MEDLINE(R) Epub Ahead of Print and In-Process & Other Non-Indexed Citations May 29, 2018

#	Searches	Results
1	exp Sleep Apnea Syndromes/ [MeSH heading changed from exp sleep disordered breathing]	0
2	exp obesity hypoventilation syndrome/	0
3	apnea/ and sleep/ [historical]	0
4	apnea/ and (hypoventilation/ or sleep disorders/) [historical]	0
5	(apn?ea adj1 attack*).mp.	3
6	(apn?ea adj1 index*).mp.	65
7	(apn?ea adj1 indices*).mp.	8
8	(apn?ea adj1 monitor*).mp.	16
9	(haddad* adj1 syndrom*).mp.	2
10	(high* altitud* adj2 (period* adj2 breath*)).mp.	3
11	(hypersomni* adj2 periodic breath*).mp.	0
12	(hypersomni* adj2 periodic respirat*).mp.	0
13	(nocturnal adj2 hypoventilation).mp.	21
14	(nocturnal adj2 hypoxemia).mp.	37

15	(obes* hypoventil* adj2 apne*?).mp.	11
16	(obes* hypoventil* adj2 apnoe*?).mp.	2
17	(obstruct* adj2 hypoapnea*).mp.	0
18	(obstruct* adj2 hypo-apnea*).mp.	0
19	(obstruct* adj2 hypoapnoea*).mp.	0
20	(obstruct* adj2 hypo-apnoea*).mp.	0
21	(obstruct* adj2 hypopnea*).mp.	89
22	(obstruct* adj2 hypopnoea*).mp.	2
23	(ondine* adj2 curse*).mp.	10
24	(ondine* adj2 syndrome*).mp.	3
25	(pickwick* adj1 syndrom*).mp.	10
26	(sleep adj2 respirat* adj1 disorder*).mp.	30
27	(sleep disorder* adj1 breathing).mp.	794
28	(sleep disorder* adj1 respirat*).mp.	20
29	(sleep* adj2 hypopn?ea*).mp.	244
30	(upper airway resistan* adj2 syndrom*).mp.	29
31	apnea-hypopnea*.mp.	970
32	apneic*.mp.	160
33	apnoea-hypopnoea*.mp.	117
34	apnoeic*.mp.	59
35	hypopneic*.mp.	6
36	hypopnoeic*.mp.	1
37	obes* hypoventil* syndrome?.mp.	83
38	osa.tw,kw.	1982
39	osahs.tw,kw.	148
40	osas.tw,kw.	407
41	sleep apne*.mp.	3672
42	sleep apnoe*.mp.	655
43	upper airway* resistan* syndrom*.mp.	30
44	or/1-43 [~~ Obstructive Sleep Apnea & Related Terms ~~]	5187
45	exp Cardiovascular Diseases/	0
46	"high blood pressur*".mp.	1464
47	(atrial adj1 fibrillat*).mp.	9211
48	(atrial adj1 flutter*).mp.	495
49	(cardiac* adj3 disease*).mp.	2837
50	(cardiac* adj3 fail*).mp.	1386
51	(cardiac* adj3 infarc*).mp.	597
52	(cardiac* adj3 illness*).mp.	42
53	(cardio* adj3 disease*).mp.	22774
54	(cardio* adj3 fail*).mp.	903
55	(cardio* adj3 illness*).mp.	87
56	(coronar* adj3 disease*).mp.	12786
57	(coronar* adj3 fail*).mp.	268

58	(coronar* adj3 illness*).mp.	13
59	(coronar* adj3 syndrom*).mp.	4256
60	(heart? adj1 attack*).mp.	566
61	(heart? adj1 failure*).mp.	17334
62	(heart? adj3 aneurysm*).mp.	45
63	(heart? adj3 arrest???).mp.	202
64	(heart? adj3 defect*).mp.	1411
65	(heart? adj3 disease?).mp.	15537
66	(heart? adj3 failure?).mp.	17435
67	(heart? adj3 infarc*).mp.	766
68	(heart? adj3 isch?em*).mp.	3154
69	(heart? adj3 neoplas*).mp.	72
70	(heart? adj3 ruptur*).mp.	68
71	(myocard* adj3 disease*).mp.	817
72	(myocard* adj3 isch?emi*).mp.	3009
73	(myocard* adj3 infarc*).mp.	15036
74	(myocard* adj3 stunning?).mp.	81
75	(pericard* adj3 effusion*).mp.	1060
76	(peripheral* adj1 arter* adj2 disease*).mp.	1901
77	(pulmonary adj2 emboli*).mp.	3637
78	(ventricular adj3 outflow* adj3 obstruct*).mp.	378
79	(ventricular adj2 dysfunct*).mp.	2113
80	angina??.mp.	3357
81	arrhythmi*.mp.	8171
82	arteriosclero*.mp.	656
83	artherosclero*.mp.	12
84	bradyarrhythmi*.mp.	155
85	brady-arrhythmi*.mp.	6
86	cardiomegal*.mp.	211
87	cardio-megal*.mp.	0
88	cardiomyopath*.mp.	7102
89	cardio-myopath*.mp.	9
90	cardiovascular.mp.	46211
91	coronary.mp.	31013
92	CVD.tw,kw.	6194
93	endo-cardi*.mp.	6
94	endocardi*.mp.	3792
95	hyperlipidaemi*5.mp.	308
96	hyperlipidemi*5.mp.	2717
97	hypertension.mp.	33493
98	hypertensive?.mp.	7413
99	myocardial*.mp.	23905
100	myocardiac*.mp.	14

101	NSTEMI.mp.	396
102	sick sinus*.mp.	148
103	STEMI.mp.	1679
104	tachycardi*.mp.	4844
105	(unstab* adj2 angina?).mp.	834
106	(valvular adj3 heart adj3 disease?).mp.	680
107	vasculopath*.mp.	1139
108	(left adj1 ventric* adj1 eject* adj1 fraction*).mp.	2510
109	lvef.mp.	1476
110	exp percutaneous coronary intervention/	0
111	angioplasty, balloon, coronary/	0
112	atherectomy, coronary/	0
113	(percutaneous* adj1 coronary adj1 intervention?).mp.	4884
114	(percutaneous* adj1 coronary adj1 revasculari*).mp.	52
115	(coronary adj1 balloon? adj1 angioplast*).mp.	33
116	(percutaneous* adj1 transluminal* adj1 coronary adj1 angioplast*).mp.	234
117	(transluminal* adj1 coronary adj1 balloon dilat*).mp.	1
118	(percutaneous* adj1 balloon? adj1 valvuloplast*).mp.	14
119	(coronary adj1 atherectom*).mp.	34
120	(rotational* adj1 atherectom*).mp.	121
121	or/45-120 [~~ Cardiac or Cardiovascular Diseases or PCI ~~]	154130
122	44 and 121 [OSA + (Cardiac or CV Diseases or PCI)]	1456
123	Inpatients/	0
124	exp Hospitalization/ or "length of stay"/	0
125	Patient admission/ or patient discharge/ or patient handoff/ or patient readmission/ or patient transfer/	0
126	intensive care units/ or coronary care units/	0
127	Critical Care/	0
128	Critical Illness/	0
129	exp Life Support Care/	0
130	inpatient?.mp.	11410
131	hospitalise?.mp.	1316
132	hospitalising.mp.	0
133	hospitalisation?.mp.	2001
134	hospitalize?.mp.	9537
135	hospitalizing.mp.	12
136	hospitalization?.mp.	15109
137	inhospital.mp.	148
138	"in hospital".ab. /freq=2	2566
139	(hospital? adj6 (stay or stays or stayed or staying)).mp.	12380
140	(length* adj6 (stay or stays or stayed or staying)).mp.	10507
141	(patient? adj6 (stay or stays or stayed or staying)).mp.	3760
142	exp Hospital Departments/	0
143	Patient Care/	0

144	Continuity of Patient Care/	0
145	Subacute Care/	0
146	(acute adj1 care adj3 unit?).mp.	93
147	(acute?? adj2 ill*).mp.	1302
148	(coronary care adj3 unit?).mp.	228
149	(critical care adj3 unit?).mp.	371
150	(intensive care adj3 unit?).mp.	13383
151	(subacute?? adj2 ill*).mp.	7
152	(ward or wards).mp.	6615
153	(cardiac care adj3 unit?).mp.	80
154	CICU.mp.	39
155	CVICU.mp.	5
156	ICU.mp.	7505
157	MSICU.mp.	5
158	TNICU.mp.	0
159	(admit or admits or admitted or admitting).mp.	24377
160	(admission or admissions).mp.	22773
161	readmit*.mp.	887
162	readmis*.mp.	3868
163	(critical?? adj2 ill*).mp.	5885
164	(acute adj2 care).mp.	2862
165	exp Hospitals/	0
166	exp Hospital Units/	0
167	patient.ab. /freq=2 or patients.ab. /freq=2 or (patient or patients).ti.	455343
168	(hospital* or department? or ward or wards or room or rooms or care unit or care units or undergo*).mp.	268505
169	167 and 168	123530
170	or/123-166,169	169408
171	122 and 170	239
172	limit 171 to english language	232
173	remove duplicates from 172	231

Embase

Embase Classic+Embase 1947 to 2018 May 30

#	Searches	Results
1	sleep disordered breathing/ [Embase]	34614
2	upper airway resistance syndrome/ [Embase]	297
3	exp Sleep Apnea Syndromes/ [MeSH heading changed from exp sleep disordered breathing]	37329
4	exp obesity hypoventilation syndrome/	1655
5	apnea/ and sleep/ [historical]	3136
6	apnea/ and (hypoventilation/ or sleep disorders/) [historical]	882

7	(apn?ea adj1 attack*).mp.	124
8	(apn?ea adj1 index*).mp.	1694
9	(apn?ea adj1 indices*).mp.	108
10	(apn?ea adj1 monitor*).mp.	2643
11	(haddad* adj1 syndrom*).mp.	40
12	(high* altitud* adj2 (period* adj2 breath*)).mp.	21
13	(hypersomni* adj2 periodic breath*).mp.	23
14	(hypersomni* adj2 periodic respirat*).mp.	14
15	(nocturnal adj2 hypoventilation).mp.	404
16	(nocturnal adj2 hypoxemia).mp.	685
17	(obes* hypoventil* adj2 apne*?).mp.	70
18	(obes* hypoventil* adj2 apnoe*?).mp.	19
19	(obstruct* adj2 hypoapnea*).mp.	2
20	(obstruct* adj2 hypo-apnea*).mp.	0
21	(obstruct* adj2 hypoapnoea*).mp.	1
22	(obstruct* adj2 hypo-apnoea*).mp.	0
23	(obstruct* adj2 hypopnea*).mp.	655
24	(obstruct* adj2 hypopnoea*).mp.	150
25	(ondine* adj2 curse*).mp.	330
26	(ondine* adj2 syndrome*).mp.	169
27	(pickwick* adj1 syndrom*).mp.	501
28	(sleep adj2 respirat* adj1 disorder*).mp.	456
29	(sleep disorder* adj1 breathing).mp.	37668
30	(sleep disorder* adj1 respirat*).mp.	168
31	(sleep* adj2 hypopn?ea*).mp.	3074
32	(upper airway resistan* adj2 syndrom*).mp.	528
33	apnea-hypopnea*.mp.	16181
34	apneic*.mp.	3642
35	apnoea-hypopnoea*.mp.	2012
36	apnoeic*.mp.	1216
37	hypopneic*.mp.	111
38	hypopnoeic*.mp.	28
39	obes* hypoventil* syndrome?.mp.	1836
40	osa.tw,kw.	21605
41	osahs.tw,kw.	1760
42	osas.tw,kw.	6424
43	sleep apne*.mp.	56780
44	sleep apnoe*.mp.	9281
45	upper airway* resistan* syndrom*.mp.	549
46	or/1-45 [~~ Obstructive Sleep Apnea & Related Terms ~~]	80301
47	exp cardiovascular disease/ [Embase]	3951790

48	coronary atherectomy/ [Embase]	76
49	exp percutaneous coronary intervention/ [Embase]	87636
50	exp Cardiovascular Diseases/	3951790
51	"high blood pressur*".mp.	20413
52	(atrial adj1 fibrillat*).mp.	114986
53	(atrial adj1 flutter*).mp.	8898
54	(cardiac* adj3 disease*).mp.	40224
55	(cardiac* adj3 fail*).mp.	29192
56	(cardiac* adj3 infarc*).mp.	10811
57	(cardiac* adj3 illness*).mp.	772
58	(cardio* adj3 disease*).mp.	369234
59	(cardio* adj3 fail*).mp.	13070
60	(cardio* adj3 illness*).mp.	1174
61	(coronar* adj3 disease*).mp.	292733
62	(coronar* adj3 fail*).mp.	4986
63	(coronar* adj3 illness*).mp.	197
64	(coronar* adj3 syndrom*).mp.	64316
65	(heart? adj1 attack*).mp.	7376
66	(heart? adj1 failure*).mp.	357166
67	(heart? adj3 aneurysm*).mp.	8983
68	(heart? adj3 arrest???).mp.	66029
69	(heart? adj3 defect*).mp.	65984
70	(heart? adj3 disease?).mp.	410502
71	(heart? adj3 failure?).mp.	377830
72	(heart? adj3 infarc*).mp.	339377
73	(heart? adj3 isch?em*).mp.	228808
74	(heart? adj3 neoplas*).mp.	1260
75	(heart? adj3 ruptur*).mp.	5576
76	(myocard* adj3 disease*).mp.	18949
77	(myocard* adj3 isch?emi*).mp.	61734
78	(myocard* adj3 infarc*).mp.	283486
79	(myocard* adj3 stunning?).mp.	2012
80	(pericard* adj3 effusion*).mp.	26400
81	(peripheral* adj1 arter* adj2 disease*).mp.	22396
82	(pulmonary adj2 emboli*).mp.	55786
83	(ventricular adj3 outflow* adj3 obstruct*).mp.	4719
84	(ventricular adj2 dysfunct*).mp.	37818
85	angina??.mp.	113769
86	arrhythmi*.mp.	202655
87	arteriosclero*.mp.	54785
88	artherosclero*.mp.	557

89	bradyarrhythmi*.mp.	2449
90	brady-arrhythmi*.mp.	135
91	cardiomegal*.mp.	19363
92	cardio-megal*.mp.	8
93	cardiomyopath*.mp.	136911
94	cardio-myopath*.mp.	169
95	cardiovascular.mp.	919419
96	coronary.mp.	674136
97	CVD.tw,kw.	44693
98	endo-cardi*.mp.	50
99	endocardi*.mp.	81344
100	hyperlipidaemi*5.mp.	6690
101	hyperlipidemi*5.mp.	79596
102	hypertension.mp.	827735
103	hypertensive?.mp.	179701
104	myocardial*.mp.	476760
105	myocardiac*.mp.	688
106	NSTEMI.mp.	5912
107	sick sinus*.mp.	5221
108	STEMI.mp.	23119
109	tachycardi*.mp.	153985
110	(unstab* adj2 angina?).mp.	27117
111	(valvular adj3 heart adj3 disease?).mp.	27870
112	vasculopath*.mp.	16399
113	(left adj1 ventric* adj1 eject* adj1 fraction*).mp.	75500
114	lvef.mp.	32577
115	exp percutaneous coronary intervention/	87636
116	angioplasty, balloon, coronary/	25928
117	atherectomy, coronary/	76
118	(percutaneous* adj1 coronary adj1 intervention?).mp.	70673
119	(percutaneous* adj1 coronary adj1 revasculari*).mp.	858
120	(coronary adj1 balloon? adj1 angioplast*).mp.	609
121	(percutaneous* adj1 transluminal* adj1 coronary adj1 angioplast*).mp.	8173
122	(transluminal* adj1 coronary adj1 balloon dilat*).mp.	4
123	(percutaneous* adj1 balloon? adj1 valvuloplast*).mp.	328
124	(coronary adj1 atherectom*).mp.	994
125	(rotational* adj1 atherectom*).mp.	1505
126	or/47-125 [~~ Cardiac or Cardiovascular Diseases or PCI ~~]	4513093
127	46 and 126 [OSA + (Cardiac or CV Diseases or PCI)]	30873
128	hospital patient/ [Embase]	142201
129	aged hospital patient/ [Embase]	729

130	hospital discharge/ [Embase]	100204
131	clinical handover/ [Embase]	1244
132	intensive care/ [Embase]	115529
133	intensive care unit/ [Embase]	130059
134	surgical intensive care unit/ [Embase]	823
135	coronary care unit/ [Embase]	10247
136	cardiac surgery intensive care unit/ [Embase]	180
137	exp hospital/ [Embase]	1081249
138	hospital department/ [Embase]	24371
139	"hospital subdivisions and components"/ [Embase]	11988
140	high dependency unit/ [Embase]	179
141	observation unit/ [Embase]	711
142	exp surgical ward/ [Embase]	5967
143	ward/ [Embase]	23309
144	Inpatients/	114983
145	exp Hospitalization/ or "length of stay"/	428540
146	Patient admission/ or patient discharge/ or patient handoff/ or patient readmission/ or patient transfer/	263834
147	intensive care units/ or coronary care units/	100660
148	Critical Care/	93622
149	Critical Illness/	26056
150	exp Life Support Care/	1517520
151	inpatient?.mp.	146531
152	hospitalise?.mp.	16424
153	hospitalising.mp.	32
154	hospitalisation?.mp.	26874
155	hospitalize?.mp.	137950
156	hospitalizing.mp.	239
157	hospitalization?.mp.	389405
158	inhospital.mp.	4903
159	"in hospital".ab. /freq=2	31796
160	(hospital? adj6 (stay or stays or stayed or staying)).mp.	144477
161	(length* adj6 (stay or stays or stayed or staying)).mp.	183076
162	(patient? adj6 (stay or stays or stayed or staying)).mp.	52517
163	exp Hospital Departments/	24371
164	Patient Care/	261138
165	Continuity of Patient Care/	232607
166	Subacute Care/	360
167	(acute adj1 care adj3 unit?).mp.	1210
168	(acute?? adj2 ill*).mp.	18170
169	(coronary care adj3 unit?).mp.	12906
170	(critical care adj3 unit?).mp.	4772

171	(intensive care adj3 unit?).mp.	190625
172	(subacute?? adj2 ill*).mp.	124
173	(ward or wards).mp.	200078
174	(cardiac care adj3 unit?).mp.	954
175	CICU.mp.	597
176	CVICU.mp.	172
177	ICU.mp.	92755
178	MSICU.mp.	34
179	TNICU.mp.	6
180	(admit or admits or admitted or admitting).mp.	320511
181	(admission or admissions).mp.	404518
182	readmit*.mp.	11851
183	readmis*.mp.	53115
184	(critical?? adj2 ill*).mp.	80701
185	(acute adj2 care).mp.	32345
186	exp Hospitals/	1081249
187	exp Hospital Units/	474426
188	patient.ab. /freq=2 or patients.ab. /freq=2 or (patient or patients).ti.	6309082
189	(hospital* or department? or ward or wards or room or rooms or care unit or care units or undergo*).mp.	3529794
190	188 and 189	1764629
191	or/128-187,190	4206366
192	127 and 191 [OSA + (Cardiac or CV Diseases or PCI) + Inpatients]	9464
193	limit 192 to conference abstract status	4201
194	limit 192 to (abstract report or books or "book review" or chapter or conference abstract or "conference review" or (book or book series or conference proceeding or trade journal))	4234
195	193 or 194	4234
196	192 not 195	5230
197	limit 196 to english language	4831
198	(exp animals/ or exp animal experimentation/ or nonhuman/) not ((exp animals/ or exp animal experimentation/ or nonhuman/) and exp human/)	6725984
199	197 not 198	4819
200	limit 197 to human	4735
201	199 or 200	4819
202	limit 201 to (embryo <first trimester> or infant <to one year> or child <unspecified age> or preschool child <1 to 6 years> or school child <7 to 12 years> or adolescent <13 to 17 years>)	440
203	201 not 202	4379
204	limit 201 to (adult <18 to 64 years> or aged <65+ years>)	2810
205	203 or 204	4532
206	remove duplicates from 205	4399

CCTR

EBM Reviews - Cochrane Central Register of Controlled Trials April 2018

#	Searches	Results
1	sleep disordered breathing/ [Embase]	481
2	upper airway resistance syndrome/ [Embase]	0
3	exp Sleep Apnea Syndromes/ [MeSH heading changed from exp sleep disordered breathing]	1572
4	exp obesity hypoventilation syndrome/	20
5	apnea/ and sleep/ [historical]	9
6	apnea/ and (hypoventilation/ or sleep disorders/) [historical]	5
7	(apn?ea adj1 attack*).mp.	9
8	(apn?ea adj1 index*).mp.	234
9	(apn?ea adj1 indices*).mp.	9
10	(apn?ea adj1 monitor*).mp.	427
11	(haddad* adj1 syndrom*).mp.	0
12	(high* altitud* adj2 (period* adj2 breath*)).mp.	9
13	(hypersomni* adj2 periodic breath*).mp.	0
14	(hypersomni* adj2 periodic respirat*).mp.	0
15	(nocturnal adj2 hypoventilation).mp.	36
16	(nocturnal adj2 hypoxemia).mp.	40
17	(obes* hypoventil* adj2 apne*?).mp.	4
18	(obes* hypoventil* adj2 apnoe*?).mp.	0
19	(obstruct* adj2 hypoapnea*).mp.	0
20	(obstruct* adj2 hypo-apnea*).mp.	0
21	(obstruct* adj2 hypoapnoea*).mp.	0
22	(obstruct* adj2 hypo-apnoea*).mp.	0
23	(obstruct* adj2 hypopnea*).mp.	52
24	(obstruct* adj2 hypopnoea*).mp.	17
25	(ondine* adj2 curse*).mp.	2
26	(ondine* adj2 syndrome*).mp.	1
27	(pickwick* adj1 syndrom*).mp.	4
28	(sleep adj2 respirat* adj1 disorder*).mp.	37
29	(sleep disorder* adj1 breathing).mp.	1561
30	(sleep disorder* adj1 respirat*).mp.	11
31	(sleep* adj2 hypopn?ea*).mp.	332
32	(upper airway resistan* adj2 syndrom*).mp.	9
33	apnea-hypopnea*.mp.	1350
34	apneic*.mp.	227
35	apnoea-hypopnoea*.mp.	302
36	apnoeic*.mp.	105
37	hypopneic*.mp.	9
38	hypopnoeic*.mp.	3
39	obes* hypoventil* syndrome?.mp.	86
40	osa.tw,kw.	1755

41	osahs.tw,kw.	128
42	osas.tw,kw.	412
43	sleep apne*.mp.	3918
44	sleep apnoe*.mp.	880
45	upper airway* resistan* syndrom*.mp.	10
46	or/1-45 [~~ Obstructive Sleep Apnea & Related Terms ~~]	5399
47	exp cardiovascular disease/ [Embase]	78972
48	coronary atherectomy/ [Embase]	116
49	exp percutaneous coronary intervention/ [Embase]	4843
50	exp Cardiovascular Diseases/	78972
51	"high blood pressur*".mp.	1001
52	(atrial adj1 fibrillat*).mp.	7966
53	(atrial adj1 flutter*).mp.	560
54	(cardiac* adj3 disease*).mp.	1638
55	(cardiac* adj3 fail*).mp.	1597
56	(cardiac* adj3 infarc*).mp.	1224
57	(cardiac* adj3 illness*).mp.	48
58	(cardio* adj3 disease*).mp.	18180
59	(cardio* adj3 fail*).mp.	1342
60	(cardio* adj3 illness*).mp.	78
61	(coronar* adj3 disease*).mp.	19289
62	(coronar* adj3 fail*).mp.	557
63	(coronar* adj3 illness*).mp.	14
64	(coronar* adj3 syndrom*).mp.	4526
65	(heart? adj1 attack*).mp.	465
66	(heart? adj1 failure*).mp.	18206
67	(heart? adj3 aneurysm*).mp.	87
68	(heart? adj3 arrest???).mp.	2330
69	(heart? adj3 defect*).mp.	1108
70	(heart? adj3 disease?).mp.	15446
71	(heart? adj3 failure?).mp.	18597
72	(heart? adj3 infarc*).mp.	8430
73	(heart? adj3 isch?em*).mp.	5941
74	(heart? adj3 neoplas*).mp.	88
75	(heart? adj3 ruptur*).mp.	53
76	(myocard* adj3 disease*).mp.	1044
77	(myocard* adj3 isch?emi*).mp.	4979
78	(myocard* adj3 infarc*).mp.	21651
79	(myocard* adj3 stunning?).mp.	99
80	(pericard* adj3 effusion*).mp.	443
81	(peripheral* adj1 arter* adj2 disease*).mp.	1838
82	(pulmonary adj2 emboli*).mp.	2438
83	(ventricular adj3 outflow* adj3 obstruct*).mp.	65

84	(ventricular adj2 dysfunct*).mp.	3269
85	angina??.mp.	10082
86	arrhythmi*.mp.	8748
87	arteriosclero*.mp.	1541
88	artherosclero*.mp.	10
89	bradyarrhythmi*.mp.	116
90	brady-arrhythmi*.mp.	2
91	cardiomegal*.mp.	221
92	cardio-megal*.mp.	0
93	cardiomyopath*.mp.	2793
94	cardio-myopath*.mp.	3
95	cardiovascular.mp.	44552
96	coronary.mp.	39882
97	CVD.tw,kw.	3046
98	endo-cardi*.mp.	3
99	endocardi*.mp.	809
100	hyperlipidaemi*5.mp.	488
101	hyperlipidemi*5.mp.	3977
102	hypertension.mp.	42763
103	hypertensive?.mp.	13946
104	myocardial*.mp.	29787
105	myocardiac*.mp.	16
106	NSTEMI.mp.	282
107	sick sinus*.mp.	231
108	STEMI.mp.	2084
109	tachycardi*.mp.	6303
110	(unstab* adj2 angina?).mp.	2655
111	(valvular adj3 heart adj3 disease?).mp.	376
112	vasculopath*.mp.	349
113	(left adj1 ventric* adj1 eject* adj1 fraction*).mp.	5404
114	lvef.mp.	2635
115	exp percutaneous coronary intervention/	4843
116	angioplasty, balloon, coronary/	3450
117	atherectomy, coronary/	116
118	(percutaneous* adj1 coronary adj1 intervention?).mp.	6552
119	(percutaneous* adj1 coronary adj1 revasculari*).mp.	168
120	(coronary adj1 balloon? adj1 angioplast*).mp.	3467
121	(percutaneous* adj1 transluminal* adj1 coronary adj1 angioplast*).mp.	756
122	(transluminal* adj1 coronary adj1 balloon dilat*).mp.	0
123	(percutaneous* adj1 balloon? adj1 valvuloplast*).mp.	4
124	(coronary adj1 atherectom*).mp.	135
125	(rotational* adj1 atherectom*).mp.	93
126	or/47-125 [~~ Cardiac or Cardiovascular Diseases or PCI ~~]	175237

127	46 and 126 [OSA + (Cardiac or CV Diseases or PCI)]	1321
128	hospital patient/ [Embase]	2
129	aged hospital patient/ [Embase]	0
130	hospital discharge/ [Embase]	1
131	clinical handover/ [Embase]	19
132	intensive care/ [Embase]	3
133	intensive care unit/ [Embase]	1
134	surgical intensive care unit/ [Embase]	0
135	coronary care unit/ [Embase]	140
136	cardiac surgery intensive care unit/ [Embase]	0
137	exp hospital/ [Embase]	3189
138	hospital department/ [Embase]	51
139	"hospital subdivisions and components"/ [Embase]	0
140	high dependency unit/ [Embase]	0
141	observation unit/ [Embase]	0
142	exp surgical ward/ [Embase]	0
143	ward/ [Embase]	0
144	Inpatients/	794
145	exp Hospitalization/ or "length of stay"/	11939
146	Patient admission/ or patient discharge/ or patient handoff/ or patient readmission/ or patient transfer/	2469
147	intensive care units/ or coronary care units/	2047
148	Critical Care/	1543
149	Critical Illness/	1308
150	exp Life Support Care/	139
151	inpatient?.mp.	9809
152	hospitalise?.mp.	1060
153	hospitalising.mp.	1
154	hospitalisation?.mp.	1999
155	hospitalize?.mp.	8302
156	hospitalizing.mp.	13
157	hospitalization?.mp.	23010
158	inhospital.mp.	273
159	"in hospital".ab. /freq=2	20037
160	(hospital? adj6 (stay or stays or stayed or staying)).mp.	14037
161	(length* adj6 (stay or stays or stayed or staying)).mp.	15780
162	(patient? adj6 (stay or stays or stayed or staying)).mp.	6197
163	exp Hospital Departments/	3083
164	Patient Care/	120
165	Continuity of Patient Care/	536
166	Subacute Care/	10
167	(acute adj1 care adj3 unit?).mp.	94
168	(acute?? adj2 ill*).mp.	1135
169	(coronary care adj3 unit?).mp.	518

170	(critical care adj3 unit?).mp.	207
171	(intensive care adj3 unit?).mp.	11602
172	(subacute?? adj2 ill*).mp.	3
173	(ward or wards).mp.	7673
174	(cardiac care adj3 unit?).mp.	42
175	CICU.mp.	20
176	CVICU.mp.	6
177	ICU.mp.	6347
178	MSICU.mp.	1
179	TNICU.mp.	0
180	(admit or admits or admitted or admitting).mp.	12084
181	(admission or admissions).mp.	17786
182	readmit*.mp.	423
183	readmis*.mp.	3783
184	(critical?? adj2 ill*).mp.	4847
185	(acute adj2 care).mp.	1793
186	exp Hospitals/	3189
187	exp Hospital Units/	3429
188	patient.ab. /freq=2 or patients.ab. /freq=2 or (patient or patients).ti.	519351
189	(hospital* or department? or ward or wards or room or rooms or care unit or care units or undergo*).mp.	184993
190	188 and 189	133424
191	or/128-187,190	168109
192	127 and 191 [OSA + (Cardiac or CV Diseases or PCI) + Inpatients]	294
193	limit 192 to english language	278
194	confer*.so.	56214
195	limit 193 to (conference or congresses or news)	44
196	194 or 195	56258
197	193 not 196	199
198	limit 197 to (cochrane childhood cancer group or cochrane neonatal group)	4
199	(paedia* or child* or infant* or neonat* or pediatr* or teen*).jw.	28191
200	198 or 199	28192
201	197 not 200	190
202	remove duplicates from 201	182

CDSR

EBM Reviews - Cochrane Database of Systematic Reviews 2005 to May 23, 2018

#	Searches	Results
1	(apn?ea adj1 attack*).ti,ab.	0
2	(apn?ea adj1 index*).ti,ab.	0
3	(apn?ea adj1 indices*).ti,ab.	0
4	(apn?ea adj1 monitor*).ti,ab.	0
5	(haddad* adj1 syndrom*).ti,ab.	0
6	(high* altitud* adj2 (period* adj2 breath*)).ti,ab.	0
7	(hypersomni* adj2 periodic breath*).ti,ab.	0
8	(hypersomni* adj2 periodic respirat*).ti,ab.	0
9	(nocturnal adj2 hypoventilation).ti,ab.	0
10	(nocturnal adj2 hypoxemia).ti,ab.	0
11	(obes* hypoventil* adj2 apne*?).ti,ab.	0
12	(obes* hypoventil* adj2 apnoe*?).ti,ab.	0
13	(obstruct* adj2 hypoapnea*).ti,ab.	0
14	(obstruct* adj2 hypo-apnea*).ti,ab.	0
15	(obstruct* adj2 hypoapnoea*).ti,ab.	0
16	(obstruct* adj2 hypo-apnoea*).ti,ab.	0
17	(obstruct* adj2 hypopnea*).ti,ab.	0
18	(obstruct* adj2 hypopnoea*).ti,ab.	0
19	(ondine* adj2 curse*).ti,ab.	0
20	(ondine* adj2 syndrome*).ti,ab.	0
21	(pickwick* adj1 syndrom*).ti,ab.	0
22	(sleep adj2 respirat* adj1 disorder*).ti,ab.	3
23	(sleep disorder* adj1 breathing).ti,ab.	5
24	(sleep disorder* adj1 respirat*).ti,ab.	3
25	(sleep* adj2 hypopn?ea*).ti,ab.	5
26	(upper airway resistan* adj2 syndrom*).ti,ab.	0
27	apnea-hypopnea*.ti,ab.	1
28	apneic*.ti,ab.	1
29	apnoea-hypopnoea*.ti,ab.	7
30	apnoeic*.ti,ab.	2
31	hypopneic*.ti,ab.	0
32	hypopnoeic*.ti,ab.	0
33	obes* hypoventil* syndrome?.ti,ab.	1
34	osa.tw,kw.	22
35	osahs.tw,kw.	6
36	osas.tw,kw.	13
37	sleep apne*.ti,ab.	4
38	sleep apnoe*.ti,ab.	21

39	upper airway* resistan* syndrom*.ti,ab.	0
40	or/1-39 [OSA]	44
41	"high blood pressur*".ti,ab.	12
42	(atrial adj1 fibrillat*).ti,ab.	41
43	(atrial adj1 flutter*).ti,ab.	1
44	(cardiac* adj3 disease*).ti,ab.	12
45	(cardiac* adj3 fail*).ti,ab.	10
46	(cardiac* adj3 infarc*).ti,ab.	4
47	(cardiac* adj3 illness*).ti,ab.	2
48	(cardio* adj3 disease*).ti,ab.	142
49	(cardio* adj3 fail*).ti,ab.	6
50	(cardio* adj3 illness*).ti,ab.	0
51	(coronar* adj3 disease*).ti,ab.	60
52	(coronar* adj3 fail*).ti,ab.	4
53	(coronar* adj3 illness*).ti,ab.	0
54	(coronar* adj3 syndrom*).ti,ab.	18
55	(heart? adj1 attack*).ti,ab.	8
56	(heart? adj1 failure*).ti,ab.	103
57	(heart? adj3 aneurysm*).ti,ab.	0
58	(heart? adj3 arrest???).ti,ab.	0
59	(heart? adj3 defect*).ti,ab.	1
60	(heart? adj3 disease?).ti,ab.	94
61	(heart? adj3 failure?).ti,ab.	103
62	(heart? adj3 infarc*).ti,ab.	15
63	(heart? adj3 isch?em*).ti,ab.	18
64	(heart? adj3 neoplas*).ti,ab.	0
65	(heart? adj3 ruptur*).ti,ab.	0
66	(myocard* adj3 disease*).ti,ab.	5
67	(myocard* adj3 isch?emi*).ti,ab.	13
68	(myocard* adj3 infarc*).ti,ab.	136
69	(myocard* adj3 stunning?).ti,ab.	0
70	(pericard* adj3 effusion*).ti,ab.	1
71	(peripheral* adj1 arter* adj2 disease*).ti,ab.	43
72	(pulmonary adj2 emboli*).ti,ab.	50
73	(ventricular adj3 outflow* adj3 obstruct*).ti,ab.	0
74	(ventricular adj2 dysfunct*).ti,ab.	3
75	angina??.ti,ab.	39
76	arrhythmi*.ti,ab.	50
77	arteriosclero*.ti,ab.	2
78	artherosclero*.ti,ab.	0
79	bradyarrhythmi*.ti,ab.	0
80	brady-arrhythmi*.ti,ab.	0
81	cardiomegal*.ti,ab.	0

82	cardio-megal*.ti,ab.	0
83	cardiomyopath*.ti,ab.	15
84	cardio-myopath*.ti,ab.	0
85	cardiovascular.ti,ab.	330
86	coronary.ti,ab.	115
87	CVD.tw,kw.	133
88	endo-cardi*.ti,ab.	0
89	endocardi*.ti,ab.	4
90	hyperlipidaemi*5.ti,ab.	12
91	hyperlipidemi*5.ti,ab.	0
92	hypertension.ti,ab.	230
93	hypertensive?.ti,ab.	64
94	myocardial*.ti,ab.	146
95	myocardiac*.ti,ab.	0
96	NSTEMI.ti,ab.	2
97	sick sinus*.ti,ab.	1
98	STEMI.ti,ab.	3
99	tachycardi*.ti,ab.	27
100	(unstab* adj2 angina?).ti,ab.	17
101	(valvular adj3 heart adj3 disease?).ti,ab.	2
102	vasculopath*.ti,ab.	2
103	(left adj1 ventric* adj1 eject* adj1 fraction*).ti,ab.	12
104	lvef.ti,ab.	7
105	(percutaneous* adj1 coronary adj1 intervention?).ti,ab.	15
106	(percutaneous* adj1 coronary adj1 revasculari*).ti,ab.	0
107	(coronary adj1 balloon? adj1 angioplast*).ti,ab.	0
108	(percutaneous* adj1 transluminal* adj1 coronary adj1 angioplast*).ti,ab.	4
109	(transluminal* adj1 coronary adj1 balloon dilat*).ti,ab.	0
110	(percutaneous* adj1 balloon? adj1 valvuloplast*).ti,ab.	0
111	(coronary adj1 atherectom*).ti,ab.	1
112	(rotational* adj1 atherectom*).ti,ab.	1
113	or/41-112 [Cardiovascular]	920
114	40 and 113 [OSA + Cardiovascular]	9
115	inpatient?.ti,ab.	114
116	hospitalise?.ti,ab.	79
117	hospitalising.ti,ab.	0
118	hospitalisation?.ti,ab.	206
119	hospitalize?.ti,ab.	15
120	hospitalizing.ti,ab.	0
121	hospitalization?.ti,ab.	43
122	inhospital.ti,ab.	2
123	"in hospital".ab. /freq=2	316
124	(hospital? adj6 (stay or stays or stayed or staying)).ti,ab.	342

125	(length* adj6 (stay or stays or stayed or staying)).ti,ab.	258
126	(patient? adj6 (stay or stays or stayed or staying)).ti,ab.	54
127	(acute adj1 care adj3 unit?).ti,ab.	0
128	(acute?? adj2 ill*).ti,ab.	30
129	(coronary care adj3 unit?).ti,ab.	1
130	(critical care adj3 unit?).ti,ab.	3
131	(intensive care adj3 unit?).ti,ab.	148
132	(subacute?? adj2 ill*).ti,ab.	0
133	(ward or wards).ti,ab.	36
134	(cardiac care adj3 unit?).ti,ab.	0
135	CICU.ti,ab.	0
136	CVICU.ti,ab.	0
137	ICU.ti,ab.	82
138	MSICU.ti,ab.	0
139	TNICU.ti,ab.	0
140	(admit or admits or admitted or admitting).ti,ab.	73
141	(admission or admissions).ti,ab.	257
142	readmit*.ti,ab.	3
143	readmis*.ti,ab.	35
144	(critical?? adj2 ill*).ti,ab.	88
145	(acute adj2 care).ti,ab.	28
146	(patient or patients).ti,ab.	3823
147	(hospital* or department? or ward or wards or room or rooms or care unit or care units or undergo*).ti,ab.	1788
148	or/115-147 [Inpatients]	4722
149	114 and 148 [OSA + Cardiovascular + Inpatients]	5
150	limit 149 to full systematic reviews	4

PubMed-NOT-Medline

Search	Query	Items found
#3	Search ((((((apnea[tiab]) OR (apnoea[tiab]) OR ("hypersomnia periodic breathing"[tiab]) AND ("nocturnal hypoventilation"[tiab] [tiab]) OR ("nocturnal hypoxemia") OR ("obesity hypoventilation apnea"[tiab]) OR ("obesity hypoventilation apnoea"[tiab]) OR ("obesity hypoventilation syndrome"[tiab]) OR ("obesity hypoventilation syndromes"[tiab]) OR ("obstructive hypoapnea"[tiab]) OR ("obstructive hypo-apnea"[tiab]) OR ("obstructive hypoapnoea"[tiab]) OR ("obstructive hypo-apnoea"[tiab]) OR ("obstructive hypopnea"[tiab]) OR ("obstructive hypopnoea[tiab]") OR ("pickwick syndrome"[tiab]) OR ("pickwick syndromes"[tiab]) OR ("pickwickian syndrome"[tiab]) OR ("pickwickian syndromes"[tiab]) OR (PSG[tiab] AND sleep*[tiab]) OR ("sleep apnea"[tiab]) OR ("sleep apnoea"[tiab]) OR ("sleep disordered breathing"[tiab]) OR ("sleep disordered respiration"[tiab]) OR ("sleep hypopnea"[tiab]) OR ("sleep hypopneas"[tiab]) OR ("sleep hypopnoea"[tiab]) OR ("sleep hypopnoeas"[tiab]) OR ("sleep respiration disorder"[tiab]) OR ("sleep apnea"[tiab]) OR ("sleep apnoea"[tiab]) OR ("apnea-hypopnea"[tiab]) OR apneic[tiab] OR ("apnoea-hypopnoea"[tiab]) OR apnoeic[tiab] OR hypopneic[tiab] OR hypopnoeic[tiab] OR osa[tiab] OR osahs[tiab] OR osas[tiab])))) AND ((Cardiovascular Diseases OR percutaneous coronary intervention OR angioplasty, balloon, coronary OR atherectomy, coronary OR ("high blood pressure") OR (atrial[tiab] AND fibrillat*[tiab]) OR (atrial[tiab] AND flutter*[tiab]) OR (cardiac*[tiab] AND disease*[tiab]) OR (cardiac*[tiab] AND fail*[tiab]) OR (cardiac*[tiab] AND infarc*[tiab]) OR (cardiac*[tiab] AND illness*[tiab]) OR (cardio*[tiab] AND disease*[tiab]) OR (cardio*[tiab] AND fail*[tiab]) OR (cardio*[tiab] AND illness*[tiab]) OR (coronar*[tiab] AND disease*[tiab]) OR (coronar*[tiab] AND fail*[tiab]) OR (coronar*[tiab] AND illness*[tiab]) OR (coronar*[tiab] AND syndrom*[tiab]) OR (heart*[tiab] AND attack*[tiab]) OR (heart*[tiab] AND failure*[tiab]) OR (heart*[tiab] AND aneurysm*[tiab]) OR (heart*[tiab] AND arrest*[tiab]) OR (heart*[tiab] AND defect*[tiab]) OR (heart*[tiab] AND disease*[tiab]) OR (heart*[tiab] AND failure*[tiab]) OR (heart*[tiab] AND infarc*[tiab]) OR (heart*[tiab] AND ischem*[tiab]) OR (heart*[tiab] AND ischaem*[tiab]) OR (heart*[tiab] AND neoplas*[tiab]) OR (heart*[tiab] AND ruptur*[tiab]) OR (myocard*[tiab] AND disease*[tiab]) OR (myocard*[tiab] AND ischemi*[tiab]) OR (myocard*[tiab] AND ischaemi*[tiab]) OR (myocard*[tiab] AND infarc*[tiab]) OR (myocard*[tiab] AND stunning[tiab]) OR (pericard*[tiab] AND effusion[tiab]) OR (peripheral*[tiab] AND arter*[tiab] AND disease*[tiab]) OR (pulmonary[tiab] AND emboli*[tiab]) OR (ventricular[tiab] AND outflow*[tiab] AND obstruct*[tiab]) OR (ventricular[tiab] AND dysfunct*[tiab]) OR angina*[tiab] OR arrhythmi*[tiab] OR arteriosclero*[tiab] OR artherosclero*[tiab] OR bradyarrhythmi*[tiab] OR brady-arrhythmi*[tiab] OR cardiomegal*[tiab] OR cardio-megal*[tiab] OR cardiomyopath*[tiab] OR cardio-myopath*[tiab] OR cardiovascular[tiab] OR coronary[tiab] OR CVD[tiab] OR endo-cardi*[tiab] OR endocardi*[tiab] OR hyperlipidaemi*[tiab] OR hyperlipidemi*[tiab] OR hypertension[tiab] OR hypertensive*[tiab] OR myocardial*[tiab] OR myocardiac*[tiab] OR NSTEMI[tiab] OR sick sinus*[tiab] OR STEMI[tiab] OR tachycardi*[tiab] OR (unstab*[tiab] AND angina*[tiab]) OR (valvular[tiab] AND heart[tiab] AND disease*[tiab]) OR vasculopath*[tiab] OR (left[tiab] AND ventric*[tiab] AND eject*[tiab] AND fraction*[tiab]) OR lvef[tiab] OR (percutaneous*[tiab] AND coronary[tiab] AND intervention*[tiab]) OR (percutaneous*[tiab] AND coronary[tiab] AND revasculari*[tiab]) OR (coronary[tiab] AND balloon*[tiab] AND angioplast*[tiab]) OR (percutaneous*[tiab] AND transluminal*[tiab] AND coronary[tiab] AND angioplast*[tiab]) OR (transluminal*[tiab] AND coronary[tiab] AND balloon dilat*[tiab]) OR (percutaneous*[tiab] AND balloon*[tiab] AND valvuloplast*[tiab]) OR (coronary[tiab] AND atherectom*[tiab]) OR (rotational*[tiab] AND atherectom*[tiab])))) AND ((inpatient[tiab]) OR (inpatients[tiab]) OR (hospitalise*[tiab]) OR (hospitalising[tiab]) OR (hospitalisation*[tiab]) OR (hospitalize*[tiab]) OR (hospitalizing[tiab]) OR (hospitalization*[tiab]) OR (inhospital[tiab]) OR (hospital[tiab] AND (stay[tiab] OR stays[tiab] OR stayed[tiab] OR staying[tiab])) OR (length*[tiab] AND (stay[tiab] OR stays[tiab] OR stayed[tiab] OR staying[tiab])) OR (patient[tiab] AND (stay[tiab] OR stays[tiab] OR stayed[tiab] OR staying[tiab])) OR ("acute care unit"[tiab]) OR ("acute care units"[tiab]) OR ("acute illness"[tiab]) OR ("acute illnesses"[tiab]) OR ("coronary care unit" [tiab]) OR ("coronary care	241

	units" [tiab]) OR ("critical care unit" [tiab]) OR ("critical care units" [tiab]) OR ("intensive care unit" [tiab]) OR ("intensive care units" [tiab]) OR ("subacute illness" [tiab]) OR ("subacute illnesses" [tiab]) OR (ward[tiab]) OR (wards[tiab]) OR ("cardiac care unit" [tiab]) OR ("cardiac care units" [tiab]) OR (CICU[tiab]) OR (CVICU[tiab]) OR (ICU[tiab]) OR (MSICU[tiab]) OR (TNICU[tiab]) OR (admit[tiab] OR admits[tiab] OR admitted[tiab] OR admitting[tiab]) OR (admission[tiab] OR admissions[tiab]) OR (readmit*[tiab]) OR (readmis*[tiab]) OR ("critical illness"[tiab]) OR ("critical illnesses"[tiab]) OR ("acute care"[tiab]) OR ((patient[tiab] OR patients[tiab]) AND (hospital[tiab] OR department[tiab] OR ward[tiab] OR wards[tiab] OR room[tiab] OR rooms[tiab] OR "care unit"[tiab] OR "care units"[tiab] OR undergo[tiab] OR undergoing[tiab]))))) AND (((publisher[sb] NOT pubstatusnihms NOT pubstatuspmcsd NOT pmcbook) OR inprocess[sb] OR pubmednotmedline[sb] OR ((pubstatusnihms OR pubstatuspmcsd) AND publisher[sb])))	
#2	Search ((publisher[sb] NOT pubstatusnihms NOT pubstatuspmcsd NOT pmcbook) OR inprocess[sb] OR pubmednotmedline[sb] OR ((pubstatusnihms OR pubstatuspmcsd) AND publisher[sb]))	3508169
#1	Search (((((apnea[tiab]) OR (apnoea[tiab]) OR ("hypersomnia periodic breathing"[tiab]) AND ("nocturnal hypoventilation"[tiab] [tiab]) OR ("nocturnal hypoxemia") OR ("obesity hypoventilation apnea"[tiab]) OR ("obesity hypoventilation apnoea"[tiab]) OR ("obesity hypoventilation syndrome"[tiab]) OR ("obesity hypoventilation syndromes"[tiab]) OR ("obstructive hypoapnea"[tiab]) OR ("obstructive hypo-apnea"[tiab]) OR ("obstructive hypoapnoea"[tiab]) OR ("obstructive hypo-apnoea"[tiab]) OR ("obstructive hypopnea"[tiab]) OR ("obstructive hypopnoea[tiab]") OR ("pickwick syndrome"[tiab]) OR ("pickwick syndromes"[tiab]) OR ("pickwickian syndrome"[tiab]) OR ("pickwickian syndromes"[tiab]) OR (PSG[tiab] AND sleep*[tiab]) OR ("sleep apnea"[tiab]) OR ("sleep apnoea"[tiab]) OR ("sleep disordered breathing"[tiab]) OR ("sleep disordered respiration"[tiab]) OR ("sleep hypopnea"[tiab]) OR ("sleep hypopneas"[tiab]) OR ("sleep hypopnoea"[tiab]) OR ("sleep hypopnoeas"[tiab]) OR ("sleep respiration disorder"[tiab]) OR ("sleep apnea"[tiab]) OR ("sleep apnoea"[tiab]) OR ("apnea-hypopnea"[tiab]) OR apneic[tiab] OR ("apnoea-hypopnoea"[tiab]) OR apnoeic[tiab] OR hypopneic[tiab] OR hypopnoeic[tiab] OR osa[tiab] OR osahs[tiab] OR osas[tiab])))) AND ((Cardiovascular Diseases OR percutaneous coronary intervention OR angioplasty, balloon, coronary OR atherectomy, coronary OR ("high blood pressure") OR (atrial[tiab] AND fibrillat*[tiab]) OR (atrial[tiab] AND flutter*[tiab]) OR (cardiac*[tiab] AND disease*[tiab]) OR (cardiac*[tiab] AND fail*[tiab]) OR (cardiac*[tiab] AND infarc*[tiab]) OR (cardiac*[tiab] AND illness*[tiab]) OR (cardio*[tiab] AND disease*[tiab]) OR (cardio*[tiab] AND fail*[tiab]) OR (cardio*[tiab] AND illness*[tiab]) OR (coronar*[tiab] AND disease*[tiab]) OR (coronar*[tiab] AND fail*[tiab]) OR (coronar*[tiab] AND illness*[tiab]) OR (coronar*[tiab] AND syndrom*[tiab]) OR (heart*[tiab] AND attack*[tiab]) OR (heart*[tiab] AND failure*[tiab]) OR (heart*[tiab] AND aneurysm*[tiab]) OR (heart*[tiab] AND arrest*[tiab]) OR (heart*[tiab] AND defect*[tiab]) OR (heart*[tiab] AND disease*[tiab]) OR (heart*[tiab] AND failure*[tiab]) OR (heart*[tiab] AND infarc*[tiab]) OR (heart*[tiab] AND ischem*[tiab]) OR (heart*[tiab] AND ischaem*[tiab]) OR (heart*[tiab] AND neoplas*[tiab]) OR (heart*[tiab] AND ruptur*[tiab]) OR (myocard*[tiab] AND disease*[tiab]) OR (myocard*[tiab] AND ischemi*[tiab]) OR (myocard*[tiab] AND ischaemi*[tiab]) OR (myocard*[tiab] AND infarc*[tiab]) OR (myocard*[tiab] AND stunning[tiab]) OR (pericard*[tiab] AND effusion[tiab]) OR (peripheral*[tiab] AND arter*[tiab] AND disease*[tiab]) OR (pulmonary[tiab] AND emboli*[tiab]) OR (ventricular[tiab] AND outflow*[tiab] AND obstruct*[tiab]) OR (ventricular[tiab] AND dysfunct*[tiab]) OR angina*[tiab] OR arrhythmi*[tiab] OR arteriosclero*[tiab] OR artherosclero*[tiab] OR bradyarrhythmi*[tiab] OR brady-arrhythmi*[tiab] OR cardiomegal*[tiab] OR cardio-megal*[tiab] OR cardiomyopath*[tiab] OR cardio-myopath*[tiab] OR cardiovascular[tiab] OR coronary[tiab] OR CVD[tiab] OR endo-cardi*[tiab] OR endocardi*[tiab] OR hyperlipidaemi*[tiab] OR hyperlipidemi*[tiab] OR hypertension[tiab] OR hypertensive*[tiab] OR myocardial*[tiab] OR myocardiac*[tiab] OR NSTEMI[tiab] OR sick sinus*[tiab] OR STEMI[tiab] OR tachycardi*[tiab] OR (unstab*[tiab] AND angina*[tiab]) OR (valvular[tiab] AND heart[tiab] AND disease*[tiab]) OR vasculopath*[tiab] OR (left[tiab] AND ventric*[tiab] AND eject*[tiab] AND fraction*[tiab]) OR lvef[tiab] OR (percutaneous*[tiab] AND coronary[tiab] AND intervention*[tiab]) OR (percutaneous*[tiab] AND coronary[tiab] AND revasculari*[tiab]) OR (coronary[tiab] AND balloon*[tiab] AND angioplast*[tiab]) OR (percutaneous*[tiab] AND transluminal*[tiab] AND coronary[tiab] AND angioplast*[tiab]) OR (transluminal*[tiab] AND coronary[tiab] AND balloon dilat*[tiab]) OR	1666

	(percutaneous*[tiab] AND balloon*[tiab] AND valvuloplast*[tiab]) OR (coronary[tiab] AND atherectom*[tiab]) OR (rotational*[tiab] AND atherectom*[tiab])))) AND ((inpatient[tiab]) OR (inpatients[tiab]) OR (hospitalise*[tiab]) OR (hospitalising[tiab]) OR (hospitalisation*[tiab]) OR (hospitalize*[tiab]) OR (hospitalizing[tiab]) OR (hospitalization*[tiab]) OR (inhospital[tiab]) OR (hospital[tiab] AND (stay[tiab] OR stays[tiab] OR stayed[tiab] OR staying[tiab])) OR (length*[tiab] AND (stay[tiab] OR stays[tiab] OR stayed[tiab] OR staying[tiab])) OR (patient[tiab] AND (stay[tiab] OR stays[tiab] OR stayed[tiab] OR staying[tiab])) OR ("acute care unit"[tiab]) OR ("acute care units"[tiab]) OR ("acute illness"[tiab]) OR ("acute illnesses"[tiab]) OR ("coronary care unit" [tiab]) OR ("coronary care units" [tiab]) OR ("critical care unit" [tiab]) OR ("critical care units" [tiab]) OR ("intensive care unit" [tiab]) OR ("intensive care units" [tiab]) OR ("subacute illness" [tiab]) OR ("subacute illnesses" [tiab]) OR (ward[tiab]) OR (wards[tiab]) OR ("cardiac care unit" [tiab]) OR ("cardiac care units" [tiab]) OR (CICU[tiab]) OR (CVICU[tiab]) OR (ICU[tiab]) OR (MSICU[tiab]) OR (TNICU[tiab]) OR (admit[tiab] OR admits[tiab] OR admitted[tiab] OR admitting[tiab]) OR (admission[tiab] OR admissions[tiab]) OR (readmit*[tiab]) OR (readmis*[tiab]) OR ("critical illness"[tiab]) OR ("critical illnesses"[tiab]) OR ("acute care"[tiab]) OR ((patient[tiab] OR patients[tiab]) AND (hospital[tiab] OR department[tiab] OR ward[tiab] OR wards[tiab] OR room[tiab] OR rooms[tiab] OR "care unit"[tiab] OR "care units"[tiab] OR undergo[tiab] OR undergoing[tiab])))	

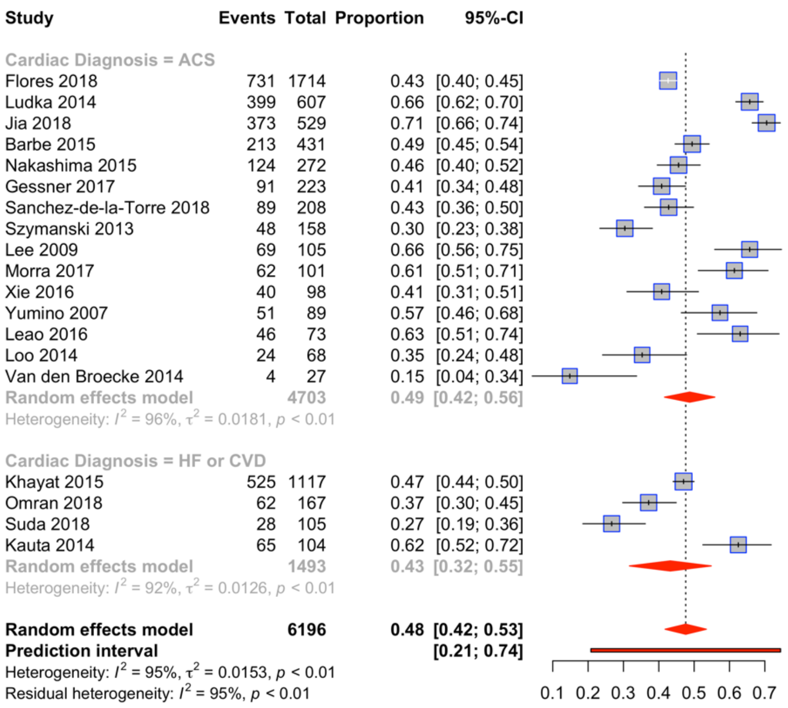

A

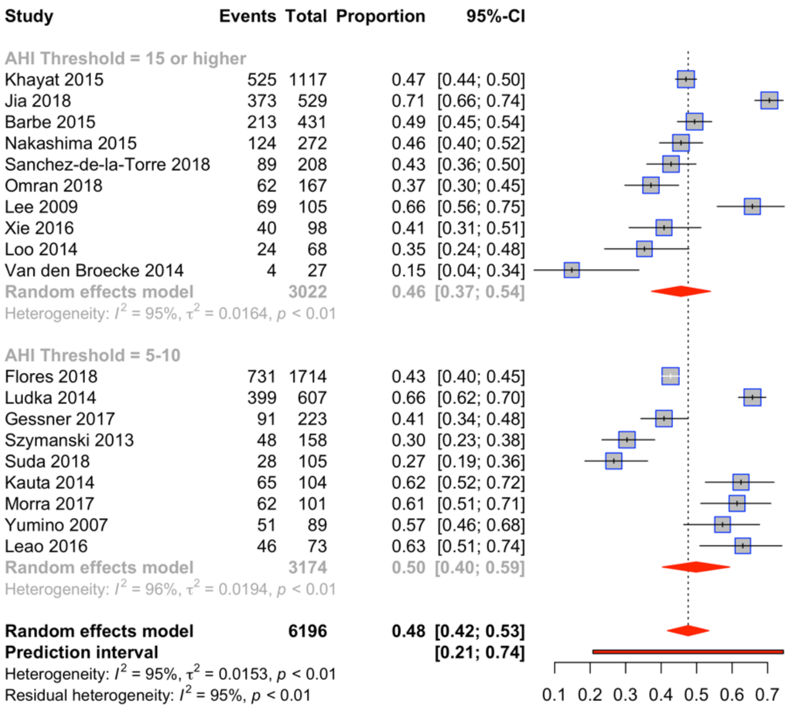

B

Supplementary Figure 1: Forrest plots of OSA prevalence among cardiac inpatients by subgroups according (A) Cardiac diagnosis and (B) AHI thresholds for OSA diagnosis.
